# Fe limitation decreases transcriptional regulation over the diel cycle in the model diatom *Thalassiosira pseudonana*

**DOI:** 10.1371/journal.pone.0222325

**Published:** 2019-09-11

**Authors:** Johanna A. L. Goldman, Megan J. Schatz, Chris T. Berthiaume, Sacha N. Coesel, Mónica V. Orellana, E. Virginia Armbrust

**Affiliations:** 1 School of Oceanography, University of Washington, Seattle, Washington, United States of America; 2 Polar Science Center, Applied Physics Laboratory, University of Washington, Seattle, Washington, United States of America; 3 Institute for Systems Biology, Seattle, Washington, United States of America; Mount Allison University, CANADA

## Abstract

Iron (Fe) is an important growth factor for diatoms and its availability is further restricted by changes in the carbonate chemistry of seawater. We investigated the physiological attributes and transcriptional profiles of the diatom *Thalassiosira pseudonana* grown on a day: night cycle under different CO_2_/pH and iron concentrations, that in combination generated available iron (Fe’) concentrations of 1160, 233, 58 and 12 pM. We found the light-dark conditions to be the main driver of transcriptional patterns, followed by Fe’ concentration and CO_2_ availability, respectively. At the highest Fe’ (1160 pM), 55% of the transcribed genes were differentially expressed between day and night, whereas at the lowest Fe’ (12 pM), only 28% of the transcribed genes displayed comparable patterns. While Fe limitation disrupts the diel expression patterns for genes in most central metabolism pathways, the diel expression of light- signaling molecules and glycolytic genes was relatively robust in response to reduced Fe’. Moreover, we identified a non-canonical splicing of transcripts encoding triose-phosphate isomerase, a key-enzyme of glycolysis, generating transcript isoforms that would encode proteins with and without an active site. Transcripts that encoded an active enzyme maintained a diel expression at low Fe’, while transcripts that encoded the non-active enzyme lost the diel expression. This work illustrates the interplay between nutrient limitation and transcriptional regulation over the diel cycle. Considering that future ocean conditions will reduce the availability of Fe in many parts of the oceans, our work identifies some of the regulatory mechanisms that may shape future ecological communities.

## Introduction

Organisms modulate their gene expression patterns to adapt their physiology to environmental conditions. Among phytoplankton, diatoms are successful in a variety of environmental conditions [1–3], both in coastal and open ocean, and across a wide range of temperature and nutrient concentrations. Previous work suggested that diatom cell division time is affected by light availability [4] and by nutrient fluctuations [5, 6], although the relationship between their photoperiod and nutrient dynamics has yet to be fully elucidated at a regulatory level. In open oceans, phytoplankton compete for macronutrients such as nitrate and phosphate, which are essential building components of these photosynthetic organisms.

Availability of iron (Fe) is an important limiting factor for the growth of phytoplankton in about 30% of the ocean [7, 8]. Large scale Fe-enrichment experiments result in blooms and the dominance of diatom populations [9]. As the pH of the ocean decreases due to higher CO_2_ concentration, availability of Fe for organisms will be further reduced due to a strengthened complexation of pH-sensitive ligands [10] and a reduced availability of carbonate ion [11]. Ocean acidification is therefore predicted to increase areas of Fe limitation in the oceans. Fe serves as a cofactor for a variety of metalloproteins, essential to both photosynthetic and respiratory pathways. Diatoms utilize numerous adaptive mechanisms to mitigate the effects of Fe limitation on growth. Strategies involve high affinity transport systems to acquire [12] or store Fe [13], a change in the ratio of electron transport chain components [14], or the replacement of cell components with Fe-free equivalents [15, 16].

Here we present a study on the effects of bioavailable Fe concentrations, CO_2_ levels and pH on the physiology and gene expression levels of the diatom *T*. *pseudonana*. We grew the diatom on a light: dark cycle, given that diatoms have adapted their cellular metabolisms to the 24-hour day-night cycle and display diel rhythms in pigment production, growth, gene expression and other basic cellular processes [17–20]. We conducted our study under light limited conditions (50 μmol photons m^-2^ sec^-1^) to assess the synergetic impact of Fe and CO_2_ on cell metabolism, while minimizing light-stress responses commonly observed under growth-limiting conditions [21]. Our results showed that light-dark conditions were the major drivers of gene expression patterns. Fe limitation disrupted the diel expression patterns for genes in most central metabolism pathways. Genes encoding light-signaling molecules however maintained a diel expression pattern throughout the different Fe concentrations and are hypothesized to play key roles in maintaining homeostasis under adverse conditions. CO_2_ levels affected carbon fixation pathways, most notably several carbonic anhydrases. By developing splice aware gene models for specific genes of interest, we also showed that alternative splicing may be an additional strategy used by diatoms to regulate expression patterns of certain proteins.

## Materials and methods

### Experimental design

Axenic *Thalassiosira pseudonana* (CCMP 1335) was grown in batch cultures using chelexed Aquil media prepared according to [22] with the addition of either 1000 nmol L^-1^ or 50 nmol L^-1^ iron (EDTA at 100 μmol L^-1^). All media and cultures were prepared in a NuAir laminar flow hood (model NU-S201-430, Plymouth, MN, USA) within a trace metal clean room. Media and bottles were sterilized via microwave and the polycarbonate bottles used for cultures and experiments were washed with 10% hydrochloric acid and rinsed with MilliQ water. Cells were grown at 20°C under a 12:12 light: dark cycle at 50 μmol photons m^-2^ sec^-1^, a light level that limits the growth rate of *T*. *pseudonana*. Prior to start of the experiments, cells were grown at the same light level and acclimated to iron-replete (1000 nM) or iron-limiting (50 nM) conditions for at least a month (~20 generations at Fe-limiting, ~40 generations at Fe-replete), but not to their respective CO_2_/pH conditions. During this pre-experiment set-up, growth rates were determined by daily monitoring of chlorophyll *a* fluorescence using a 10-AU fluorometer (Turner Designs, Sunnyvale, CA, USA). The starting cell concentration for the three experimental replicates was 30,000 cell/mL (determined by using a hemacytometer). All experimental samples were drawn from bottles via a syringe port 6 hours into the light (midday) and 6 hours into the dark phase (midnight). We used two *p*CO_2_/pH conditions of 400 ppm/8.1 and 800 ppm/7.8, in combination with two Fe additions of 1000 nM and 50 nM. The resulting concentration of available Fe (hereafter referred as Fe’) for each condition was calculated using equations from Morel and Herring [23]. We considered the four conditions as four different Fe’ concentrations for the rest of the analysis. All experiments were performed in triplicate.

### *p*CO_2_ adjustments

The media was bubbled with air containing 400 or 800 ppm CO_2_ prior to cell inoculation. The two CO_2_ levels were generated by first stripping the water from laboratory air with Du-cal (Drierite company, Xenia, OH, USA) and CO_2_ with Sodasorb (Divers SupplyInc., Gretna, LA, USA) and then using mass-flow controllers (model GFC-17, Aalborg, Orangeburg, NY, USA) to mix with 99.99% pure CO_2_ at precise ratios (Praxair, Danbury, CT, USA). The CO_2_ concentration of the gas was confirmed with a CO_2_ analyzer (model S151, Qubit Systems, Kingston, Ontario, Canada). Air flow at a rate of 0.4 standard liter/min to each bottle was controlled with an air flow meter (model 6A0101BV-AB, Dakota Instruments Inc., Orangeburn, NY, USA). The CO_2_-air mixture was passed through a 0.2 μm Polyvent PTFE filter (Whatman) and bubbled into 500 mL of 1M HCl to remove trace metals, and then through 1L of Milli-Q water to equilibrate pH and a second 0.2 μm filter to ensure sterility before entering the culturing 2L bottle.

### Macronutrients measurements

Triplicate samples (2mL each) for each of the three replicate bottles per treatment were filtered through a polycarbonate 0.2 μm membrane and stored frozen at -20°C until colorimetric analysis. All samples were measured in a 96 well plate with a SpectraMax M2 microplate reader (Molecular Devices, Sunnyvale, CA, USA). Nitrate samples were prepared and analyzed with Szechrome NAS (08762–5, Polysciences, Inc. Warrington, PA, USA) according to manufacturer instructions. Phosphate samples were prepared and analyzed with SensoLyte Malachite Green Phosphate Assay Kit (AS-71103, AnaSpec, Inc., Fremont, CA, USA) according to manufacturer instructions. Silicate samples were prepared and analyzed based on the methods of [24] for reactive silicate measurements. Nutrient consumption rates were calculated as the slope of nutrients concentrations during the exponential phase of the diatom growth.

### Fluorescence and Fv/Fm

*In vivo* chlorophyll *a* fluorescence was determined with a Turner 10-AU fluorometer (Turner Designs, Sunnyvale, CA, USA). Photochemical yield of photosystem II (Fv/Fm) and chlorophyll *a* concentration (Chl*a* in μg/L) were determined in triplicate with a Phyto-PAM fluorometer (Heinz Walz GmbH, Effeltrich, Gemany), calibrated with chlorophyll *a* standards (Turner Designs), after leaving samples in the dark for 15min.

### Carbonate chemistry measurements

Dissolved inorganic carbon (DIC) was measured at each time point, for each of the three replicate bottles, with an AS-C3 DIC analyzer (Apollo SciTech Inc., Bogart, GA, USA) and calibrated with certified reference materials (Andrew Dickson, UCSD). pH was measured at each time point, for each of the three replicate bottles, using m-cresol purple dye according to the protocol of Dickson et al. [25] and accuracy was checked with pH standards (Andrew Dickson, UCSD). The fCO_2_ (effective partial pressure of the gas) and alkalinity were calculated using CO_2_SYS software [26].

### Flow cytometry

Triplicate samples for each of the three replicate bottles were fixed with glutaraldehyde to a final concentration of 0.25%, for 20min in the dark, before being flash frozen and stored at -80°C until analysis. Samples were run on an Influx flow cytometer (BD Biosciences, Seattle, WA, USA) equipped with a 488nm laser of 200 mW; data collection (at 692 ± 40 nm and 580 ± 30 nm) was triggered by forward light scatter. To minimize coincidence and improve population resolution, flow rates and sample concentrations were adjusted to achieve event rates below 1000 s^-1^. One-micrometer beads were added to each sample as an internal standard (PolySciences Inc., Warring- ton, PA, USA). Volumes for each run were determined by weighing the samples before and after analysis. Flow cytometry files were processed using Flowing Software (Cell Imaging Core, Turku Centre for Biotechnology, Finland). Normalized forward scatter (fsc) were obtained by dividing mean fsc of cells by mean fsc of beads.

### RNA

Samples for transcriptomes were collected over one day during the exponential growth phase at 6 hours into the light and 6 hours into the dark phase. Approximately 3 x 10^7^ cells were gently filtered onto a 0.4 μm polycarbonate Millipore filter (HTTP04700), flash frozen, and stored at -80°C until analysis. Samples were extracted with the ToTALLY RNA Total RNA Isolation Kit (Ambion, AM1910). Library preparation and sequencing was performed at the Northwest Genomics Center (University of Washington) via Illumina NextSeq. Reads were reverse complemented during library preparation. Sequence data and gene expression are available at the NCBI Gene Expression Omnibus (GEO) under the identifier GSE131042.

### Expression analysis

Sequence reads from Illumina were trimmed with Trimmomatic 0.36 [27], run in paired-end mode with the following parameters: ILLUMINACLIP (cut adapter and other Illumina-specific sequences) was set to TruSeq3-PE.fa.2:30:10, Leading and Trailing thresholds were 25, the sliding window trimming approach was 4:25, the average quality level (AVGQUAL) was 30, and the minimum length (MINLEN) was 100. The reads were then mapped against the JGI Thaps3 FilteredModels2 genome (https://genome.jgi.doe.gov/Thaps3/Thaps3.home.html) using the sequence aligner Hisat2-2.1.0 [28] (maximum intron length: 2000, using rna-strandness option “RF” to account for the reverse complement nature of the reads, no discordant alignment). A summary table of all the read counts (raw and after quality control/trimming) is given in S1 Table for each sample. Transcript abundances were quantified with HTSeq 0.6.1 using default settings, counting the number of fragments that aligned to a given gene in the JGI Thaps3 FilteredModels2 set. The edgeR pipeline was used to perform differential transcript abundance for each gene [29]. We conducted pairwise comparisons between day samples and night samples at each Fe’ concentration by using a generalized linear model (GLM) likelihood ratio test. Unless otherwise noted, all results shown in this work consider a significant differential expression at *p*<0.01 and FDR<0.05. An enrichment analysis of genes that maintained differential expression between day and night was performed using DAVID (Database for Annotation, Visualization and Integrated Discovery) with the classification stringency set on “high”. A pathway was considered enriched if *p*<0.05. To perform a Kyoto Encyclopedia of Genes and Genomes (KEGG) pathway enrichment analysis, the JGI ID was mapped to the KEGG ID system using BlastKOALA (https://www.kegg.jp/blastkoala/). To determine the number of genes differentially expressed between the day and the night time points, we conducted a Fisher exact test for each of the 101 pathways with mapped JGI IDs and then performed a Benjamini Hochburg procedure to remove false positives (significant enrichment if the value was below 0.05). Selected KEGG pathways, as well as pathways for transcription factors [30] and signaling molecules [31] were further analyzed to look at differential expression over day: night within each pathway. Further gene curation of the KEGG pathways was done for photosynthesis, carbon fixation and glycolysis to produce complete pathways. Predictions for protein localizations in the carbon-related pathways was done following [32].

### Hierarchical clustering and multidimensional scaling

To generate hierarchical clustering of the samples, we calculated RPKM (Reads Per Kilobase Million) for each gene using the edgeR package. We retained those genes that were transcribed in at least three samples and filtered out those genes for which transcript abundance was less than 10% of the median value and then used the function hclust in R. The plotMDS function was used for multidimensional scaling, which is a graphical representation of dissimilarities between objects in as few dimensions as possible. The function converts the counts to log-counts-per-million and calculates distances between paired samples based on the log2 fold changes (log2FC) of the top 500 genes with the highest log2FC between these paired samples.

### Reference based assembly and identification of specific isoforms

Sequence alignments to the *T*. *pseudonana* genome were used as inputs to the genome-guided RNA-seq assembler Stringtie-1.3.3b [33]. An assembled transcript set was separately generated for each growth condition, with biological replicates pooled per condition. During this initial assembly, we used: the Stringtie setting “-g 1” to only allow directly adjacent reads on the same strand to be merged into the same contig, and the setting “-c 5” to require a minimum coverage of five when reporting a final contig. The assembled transcripts from all conditions were then merged into a single transcript set with the “—merge” setting of Stringtie. For this merging step we again used a “-g 1” setting to only allow contigs directly adjacent, and on the same strand, to be merged. Transcript abundances for the merged Stringtie set were separately estimated for each sample replicate with kallisto v0.43.1 [34] using 100 bootstrap samples. The “—rf-stranded” setting was used for forward sense counts and “—fr-stranded” was used for reverse sense counts, to account for the reverse complement nature of the reads. This approach allowed us to identify multiple transcript isoforms per gene, better define the 5' and 3' untranslated regions (UTRs), and identify non-canonical introns based on gapped read alignments. Differential expression analysis was done as described above using edgeR.

## Results

### Effects of iron limitation and CO_2_/pH on diatom physiology

The impacts of iron (Fe) limitation and CO_2_/pH changes were investigated for the centric diatom *Thalassiosira pseudonana*. Cells were grown at a non-saturating light intensity (50 μE m^-2^ s^-1^) on a 12:12 light: dark cycle, and subjected to two different additions of Fe (1000 nM and 50 nM) and two CO_2_/pH conditions (400 ppm/8.1 and 800 ppm/7.8). Because the availability of Fe decreases at lower pH [10], the two different additions of Fe associated with the two CO_2_/pH conditions resulted in four different available Fe concentrations, or Fe’: 1160, 233, 58 and 12 pM.

**Fig 1:**
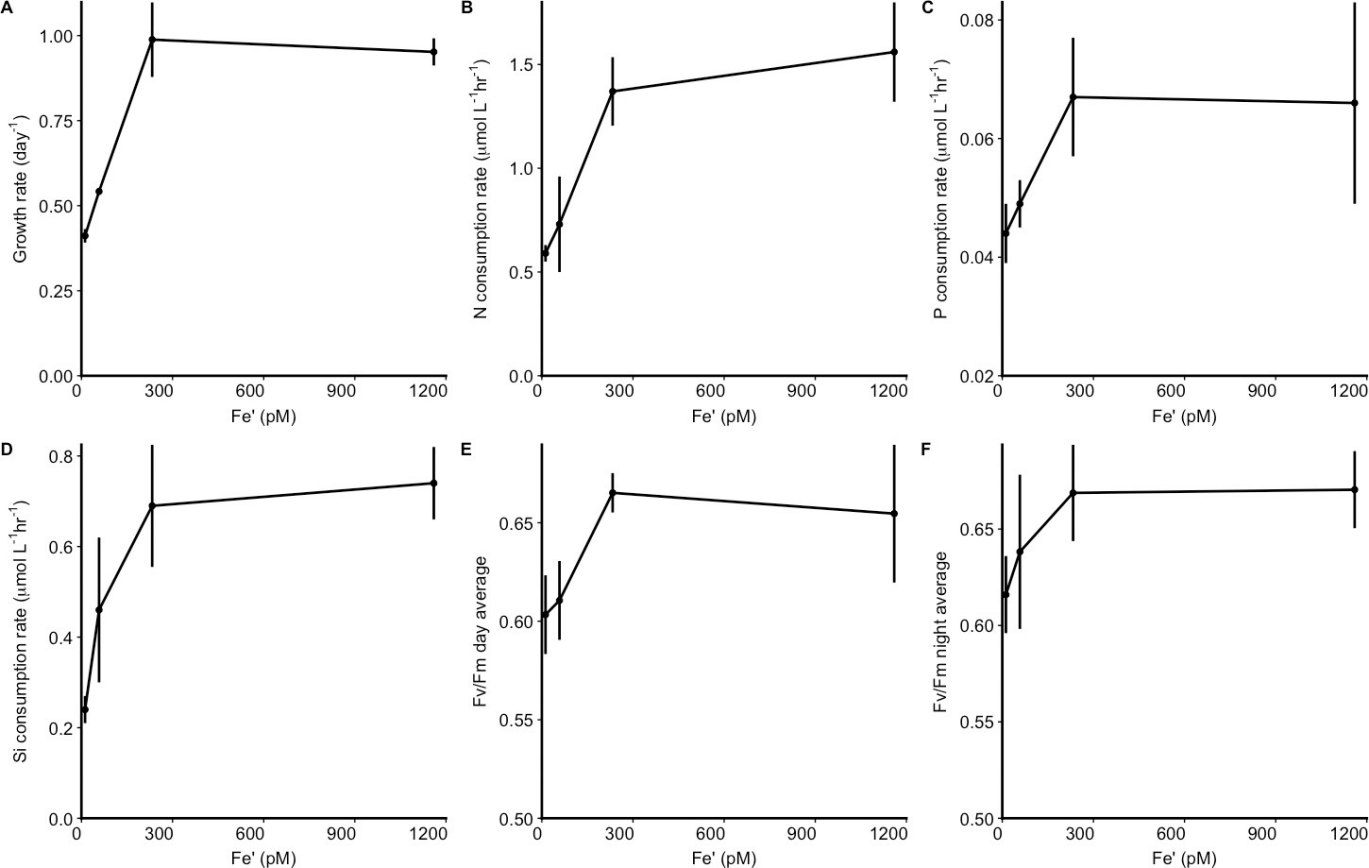
The impact of Fe’ concentrations (in pM) on different physiological parameters. (A). Growth rate (day^-1^) and (B) nitrate, (C) phosphate and (D) silicate consumption rates (in μmol L^-1^ hr^-1^), average of Fv/Fm measured during the (E) day or (F) night during exponential growth. Error bars (A-D) represent standard deviation of the triplicates. Error bars for Fv/Fm (E. F) represent the square root of the variances for each measurement used in the average.

Steady-state growth rates (S1 Fig) and nutrient (nitrate, silicate and phosphate) consumption rates (S2 Fig) increased linearly between Fe’ concentrations of 12 pM and 233 pM. Growth rates (One way Anova: [F(3,8) = 72.0, p = 3.95x10^-6^]) and nutrient consumption (NO_3_: [F(3,8) = 19.2, p = 5.17x10^-4^], Si: [F(3,8) = 14.5, p = 1.35x10^-3^]), except for phosphate consumption (PO_4_: [F(3,8) = 3.70, p = 0.062]), showed a significant effect of Fe’ for the four conditions. Post-hoc tests revealed that growth rates (*t*-test; *p* = 0.62) and nutrient consumption rates were not significantly different (*p* = 0.33 for NO_3_, *p* = 0.59 for Si) between the two highest Fe’ levels of 233 and 1160 pM (Fig 1A–1D, S2 Fig). Similarly, mean values of the photochemical yield of photosystem II (Fv/Fm) during either the day or the night showed a significant effect of Fe’ (day: [F(3,8) = 390.8, p = 5.18x10^-9^], night: [F(3,8) = 263.9, p = 2.46x10^-8^]) and also increased linearly between 12 pM and 233 pM Fe' (Fig 1E and 1F), with no significant difference between 1160 pM and 233 pM Fe' (post-hoc *t*-test: *p* = 0.07 for day average; *p* = 0.30 night). However, relative chlorophyll *a* fluorescence (Relative Fluorescence Unit or RFU) decreased at lower Fe’ (S1A and S1C and S1D Fig), indicating that Fe’ directly impacted the light harvesting system. Furthermore, CO_2_ also had a noticeable effect on the light harvesting system. The RFU per cell was modulated by CO_2_ concentration with the curves representing experiments at CO_2_ of 400 ppm (Fe’ = 1160 pM and 58 pM) at higher overall values than the two curves for experiments at 800 ppm (S1C Fig). Maximum cell yields at the end of the stationary phase showed a significant Fe’ effect ([F(3,32) = 33.5, p = 5.47x10^-10^] performed on the last three measurements for each replicate) but post-hoc *t*-tests revealed they were similar at 233 pM and 1160 pM (*p* = 0.34) and significantly higher at Fe’ 58 pM (*t*-test with Fe’ 1160 pM: p = 5.2x10^-4^) and Fe' 12 pM (*t*-test with Fe’ 58 pM: p = 3.7x10^-5^) (S1B Fig). Cell volumes decreased throughout the growth curve, as inferred from forward light scatter (S1E Fig). During stationary phase, all measurements of RFU/cell converged toward the same value, regardless of the treatment (S1C Fig) and average Fv/Fm values decreased at all Fe’ (S3 Fig).

### Transcriptional response to light-dark, CO_2_/pH and Fe concentrations

To determine the effects of Fe-limitation and CO_2_/pH changes on transcriptional profiles, samples were taken for RNA analysis from each triplicate culture at midday and midnight during exponential phase (S1A Fig). Transcripts were detected for 10,583 genes of the 11,395 total predicted thaps3 genes (https://genome.jgi.doe.gov/Thaps3/Thaps3.info.html) (S2 Table).

**Fig 2:**
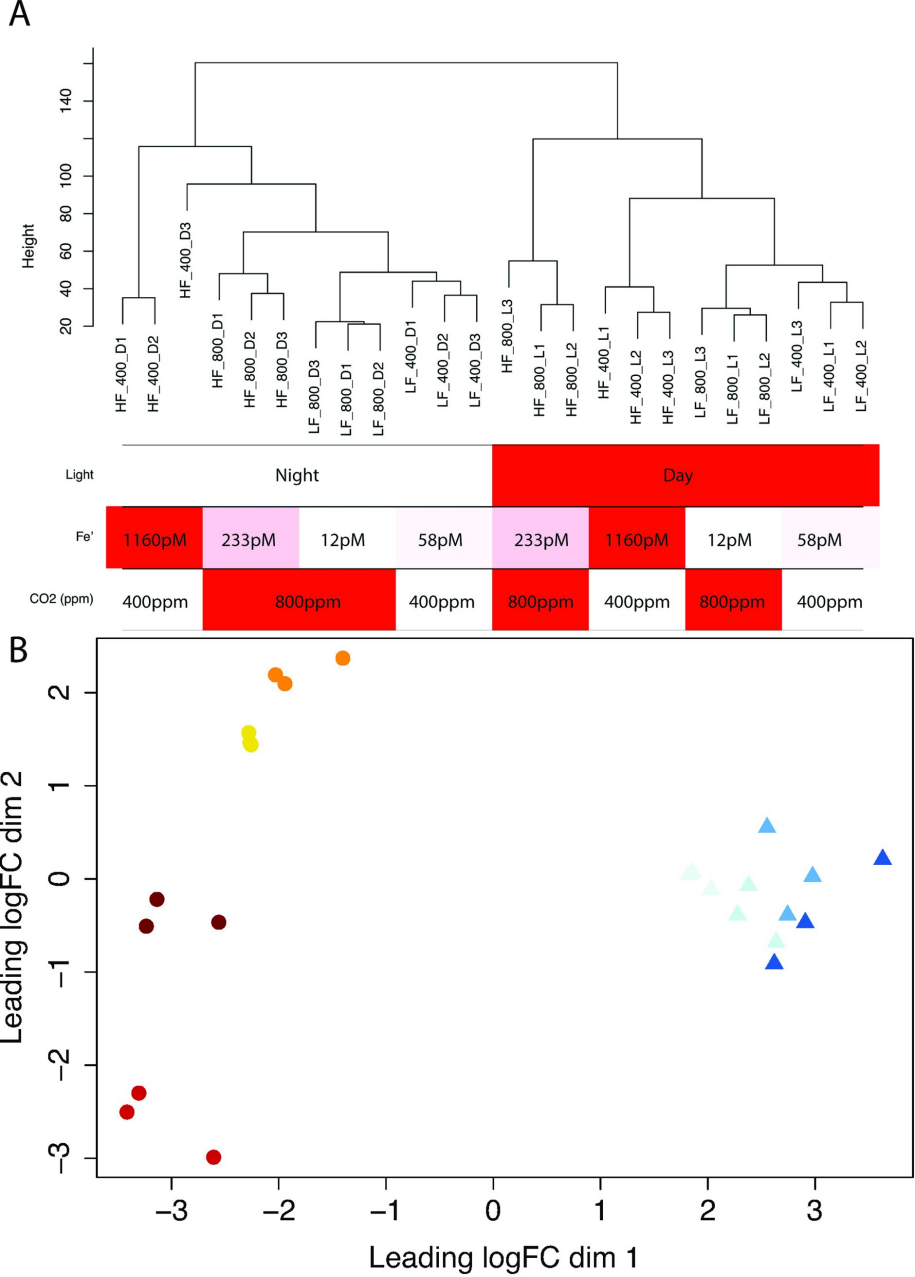
**Dendrogram representing the hierarchical clustering (A) or multidimensional scaling (MDS) (B) of the transcriptome profiles.** (A) Height in the dendrogram reflects both the order the clusters were joined and the distance between them. HF = high Fe, LF = low Fe, 400 and 800 designate the CO_2_ levels (in ppm). L and D indicates samples taken in the light (L) or in the dark (D). Red shades in table indicate Fe’ concentration (darker at 1160 pM to lighter at 12 pM), CO_2_ (red = 800 ppm; no color = 400 ppm) and sampling time (red = midday; no color = midnight). (B) Distances between samples indicate the *leading log2-fold-change* or the root-mean-square log2-fold-change between the samples for the top 500 genes that distinguish those samples, in two-dimensional space (dim 1 and dim 2). Circles represent light samples from the highest Fe’ (dark red) to the lowest Fe’ (yellow). Triangles represent dark samples from the highest Fe’ (dark blue) to the lowest Fe’ (light blue).

Hierarchical clustering of the number of reads per gene normalized for library size indicated tight replication among triplicate profiles (Fig 2A). The time of sampling–presence or absence of light–was the main influence on differences in expression patterns among the samples. Fe’ concentration drove a secondary level of clustering. The third influence on clustering was the concentration of CO_2_/pH. Samples taken during the night clustered in order of Fe’ availability, with expression profiles at 1160 pM of Fe’ most similar to those at 233 pM, and 233 pM itself more similar to the two limiting Fe’ (Fig 2A). During the day, the similarity was slightly altered, with 1160 pM samples most similar to the two deplete Fe’ samples, while samples taken at 233 pM (high CO_2_) were most dissimilar from the other Fe’ treatments.

To further assess the distances/dissimilarity between the different expression of transcription profiles, we performed a multidimensional scaling, which approximates the log2-fold-changes (log2FC) between samples (Fig 2B). We calculated pair-wise distances for the 500 genes with the highest log2FC (N.B.: 500 genes roughly corresponded to the number of differentially expressed genes with large log2FC between any 2 samples). The greatest distance is between day and night profiles. Among the day profiles, Fe’ further segregates the samples. As observed with hierarchical clustering, day samples at 233 pM (with a CO_2_ of 800 ppm) were most dissimilar to the rest of the profiles. The night profiles were more similar to each other, with no clear distances between treatments at night.

Across all Fe’, some of the most abundant transcripts during the day encoded the light harvesting proteins fucoxanthin chlorophyll *a*/*c* (S2 Table). At the two replete Fe’ (1160 and 233 pM), transcripts for the genes encoding a precursor for oxygen-evolving enhancer protein and a photosystem II protein were also among the most abundant in the light. At the two deplete Fe’ (58 and 12 pM), transcripts encoding several ABC transporters and a glyceraldehyde-3-phosphate dehydrogenase were among the most abundant in the light. A different pattern was observed during the night. At all Fe’, night-time transcript abundances were highest for the genes encoding translation elongation factor and ribosomal proteins and cytochrome b5. At the two lowest Fe’ concentrations (58 and 12 pM), night-time transcript abundances were highest for two genes that encode a heme peroxidase and a cytochrome c heme-binding site. At these Fe’ concentrations, transcripts encoding copper-induced cell-surface proteins, FTR1-iron permease-encoding genes, and several ABC transporters were among the most abundant (within the top 20 most highly transcribed genes) during both the day and the night.

### Diel transcription patterns and Fe limitation

To explore the potential impact of Fe limitation on overall day: night transcriptional patterns, differential gene expression between day and night was detected using edgeR for each Fe’ condition. A gene was considered significantly diel expressed if *p*<0.01 and FDR<0.05. We identified 8,543 genes that were differentially transcribed between day and night at one or more Fe’ level, which constituted ~80% of all detected transcripts (Table 1, S3 Table). This result confirmed the strong regulatory influence of light and dark on gene expression in this organism. At the highest Fe’, more than half (59%) of the transcribed genes were differentially transcribed between the day and night; at the lowest Fe’ of 12 pM, only about 30% of the genes were differentially transcribed (Table 1). A significant 15% reduction from 6265 to 5340 in differentially expressed genes between day and night was also observed at the two replete Fe’ concentrations of 1160 and 233 pM, despite growth rates that were not significantly different (Fig 1A). Thirty-one of the genes that lost day-night differential transcription from 1160 to 233 pM encoded transcription factors, including the photoreceptor Aureochrome 1A.

**Table 1:** Summary of number and percent of total transcribed genes (in parentheses) differentially expressed (*p*<0.01 and FDR<0.05) over the light: Dark (L:D) cycle at different Fe’ conditions.

		1160 pM	233 pM	58 pM	12 pM	At all Fe'
Significantly L:D expressed	Light	2775	2344	1688	1221	593
		(26.2)	(22.1)	(16.0)	(11.5)	(5.6)
	Dark	3490	2996	2266	1999	995
		(33.0)	(28.3)	(21.4)	(18.9)	(9.4)
	Total	6265	5340	3954	3220	1654
		(59.2)	(50.5)	(37.4)	(30.4)	(15.6)
Not significantly L:D expressed		4318	5243	6629	7363	
		(40.8)	(49.5)	(62.6)	(69.6)	

Total genes Thaps3: 11395

Total expressed genes: 10583

L:D expressed for at least 1 Fe': 8543 (80.7%)

About 1650 genes (15.6% of all detected genes) displayed differential transcript abundance between day and night at all Fe’ concentrations (Table 1). Nearly 1000 of these genes were differentially transcribed during the night, with the remainder of these genes differentially transcribed during the day, including genes encoding light harvesting proteins. These gene products were enriched in components of glycolysis (pyruvate kinase and triose-phosphate isomerase), translation (translation elongation factor, aminoacyl t-RNA synthetase), membrane components, and chlorophyll *a*-*b* binding proteins (see S4 Table) and may fulfill basal metabolic functions required over the diel cycle, regardless of Fe availability. In addition, one of the genes that displayed the greatest differential transcript abundance across Fe’ treatments was differentially expressed during the day and encoded the transcription factor bHLH3 (Thaps3_25797); two other highly expressed genes were differentially transcribed during the night and encoded heat shock factors (Thaps3_3698, Thaps3_10761) (S3 Table). In addition to these three genes, the two deplete Fe’ concentrations shared more than half of the genes in their top 20 most differentially transcribed genes, both during the day and during the night (S3 Table). These genes encoded several hypothetical proteins, a Zinc finger-containing protein (Thaps3_11947), a G-protein (Thaps3_23850), an aldose epimerase (Thaps3_31636), and an ascorbate peroxidase (Thaps3_262753).

### KEGG pathway enrichment analysis

A pathway enrichment analysis was performed to assess whether the diel expression of genes within different KEGG pathways was differentially affected by Fe limitation. Out of the 10583 expressed transcripts, 2522 were mapped to a KEGG ID, representing 101 pathways (S5 Table). At each Fe’ level, we assessed the number of genes within a given pathway that were either differentially expressed between night and day (diel) or not differentially expressed between night and day (not diel), relative to the total number of genes outside a given pathway that were or were not diel. Overall, the number of pathways enriched in diel expressed genes increased when Fe’ decreased from 1160 pM to 58 pM: 8 pathways at 1160 pM, 17 at 233 pM and 34 at 58 pM. At 12 pM the number of enriched pathways was 23. This increasing trend of enriched pathways with decreasing Fe’ is correlated with the overall decrease of total gene numbers that are diely expressed: ~1600 genes at 1160 pM, ~1400 genes at 233 pM, ~1000 genes at 58, and ~900 genes at 12 pM. Two of the most enriched KEGG pathways at the two higher Fe' concentrations are ribosome and ribosome biogenesis (S5 Table), whereas at the two lower Fe’, diel expression of genes of the Glycolysis/Gluconeogenesis pathway are among the most significantly enriched.

### Diel transcriptions of genes encoding enzymes of different metabolic pathways are disrupted by Fe limitation

**Fig 3:**
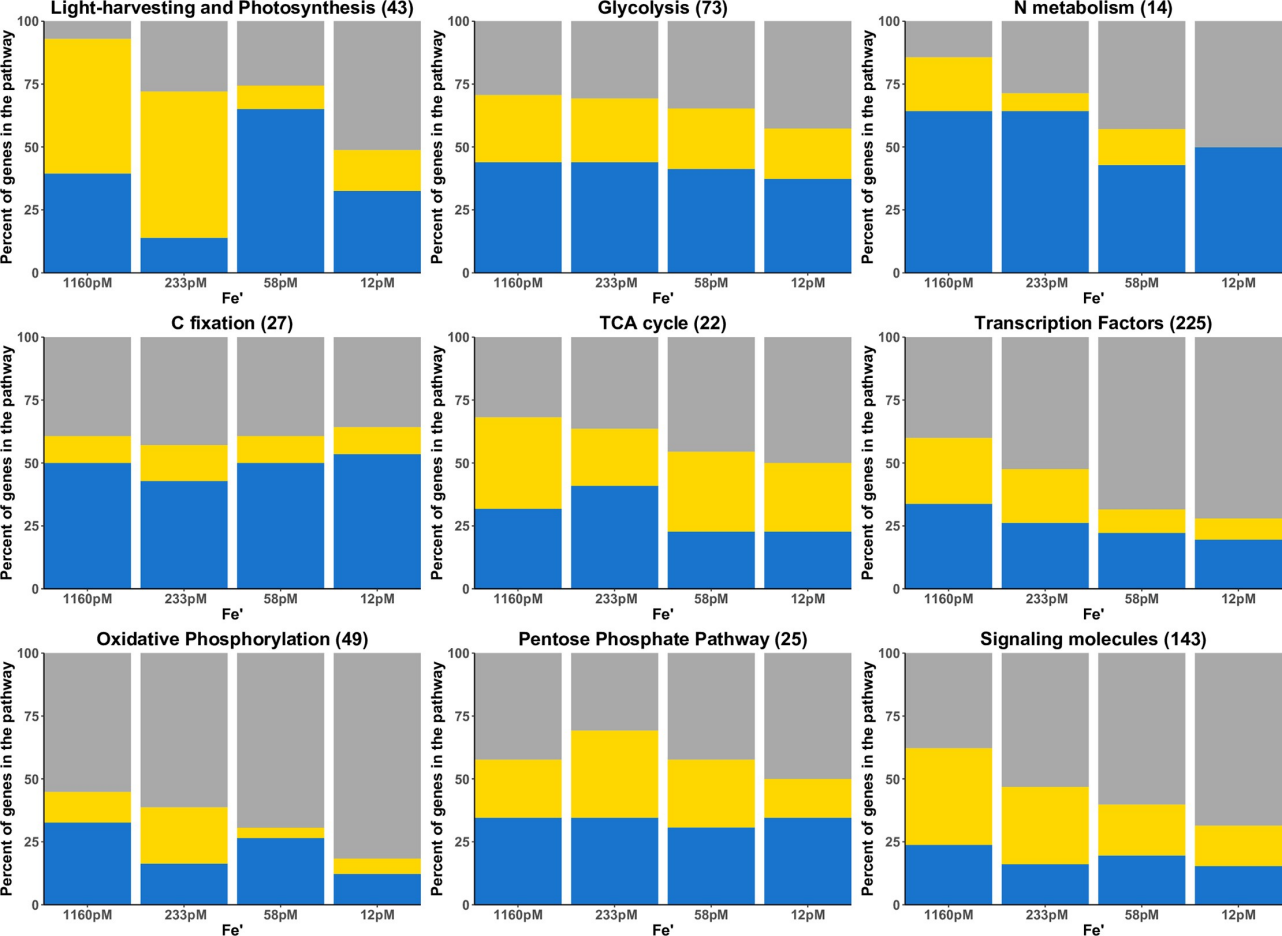
Percent of genes in KEGG-defined pathways with differential transcription between midday and midnight at different Fe’. Yellow: genes with significantly higher transcript abundance at midday (*p*<0.01 and FDR<0.05), blue: genes with significantly higher transcript abundance at midnight (*p*<0.01 and FDR<0.05), grey: genes with no significant change in transcript abundance between day-night (*p*>0.01). Numbers in parenthesis represent the total number of genes in a given pathway. List of genes for each pathway is presented in S6 Table.

We explored in more detail how Fe availability affected the diel transcription of genes encoding proteins involved in specific metabolic pathways: energy metabolism (light-harvesting and photosynthesis, carbon fixation, oxidative phosphorylation), carbohydrate metabolism (glycolysis/gluconeogenesis, TCA cycle and pentose phosphate pathway) and nitrogen metabolism (Fig 3). To do this, we manually curated three pathways to produce complete reactions: photosynthesis (to add the light-harvesting proteins in particular), carbon fixation and the pentose phosphate pathway, as these pathways were incomplete in the KEGG model. A pair-wise differential expression analysis (significance at *p*<0.01 and FDR<0.05) was conducted for each day and night sample at each Fe’ condition (Fig 3, S6 Table). This allowed us to compared the diel expression of genes within a pathway at different Fe’ levels, independently from the diel expression of the genes outside said pathway. At the highest Fe’, less than 50% of the genes involved in oxidative phosphorylation were significantly differentially expressed between day and night. This is the only pathway at 1160 pM with less than 50% of its genes significantly diely expressed at this *p* value cut off (Fig 3). A striking feature of this analysis was the lack of synchrony in transcription patterns within a given pathway, with different genes differentially transcribed either during the night or day. This difference in temporal expression of the genes within a pathway persisted at all Fe’ level for all pathways except N metabolism in which all the genes were differentially transcribed at night at the lowest Fe’.

A large proportion of genes from more than half of the examined metabolic pathways lost the differential light/dark transcript abundance patterns at lower Fe’ concentrations (Spearman correlation: -1); light-harvesting and photosynthesis, pentose phosphate pathway and carbon fixation did not follow this trend (S4A–S4G Fig). This loss of diel expression resulted from either an increase in transcript abundance at a given time when the transcripts were not normally abundant or a decrease in abundance at the time they were normally transcribed. Calculation of the RPKM of all transcripts in a pathway during day and night revealed that the loss of diel expression was, in most cases, due to the overall reduction in transcript abundance at lower Fe’ (S7 Table and S5 Fig). Interestingly, this overall reduction in transcript abundance was driven primarily by a reduction in transcription at night; transcript abundance during the day remained either constant or decreased only slightly with lower Fe’ concentrations (S5 Fig).

The number of differentially transcribed genes within the light-harvesting/photosynthetic pathway decreased with decreasing Fe’, with the exception of 58 pM which had slightly more significantly transcribed genes than at 233 pM (Fig 3, S4A Fig). At the two replete Fe’ concentrations, a high number of genes were upregulated during the day, whereas at the two deplete Fe’, most genes were upregulated during the night. In particular, genes encoding for ferredoxin/cytochrome c (Thaps3_35934), a plastoquinone reductase (Thaps3_6361), an oxygen-evolving protein precursor (Thaps3_34830) and several photosystem II proteins (Thaps3_2848, Thaps3_20603, Thaps3_24769, Thaps3_32964) that were upregulated during the day at the two replete Fe’, became more highly expressed at night at the two deplete Fe’ (S4A Fig–indicated by a *).

**Fig 4:**
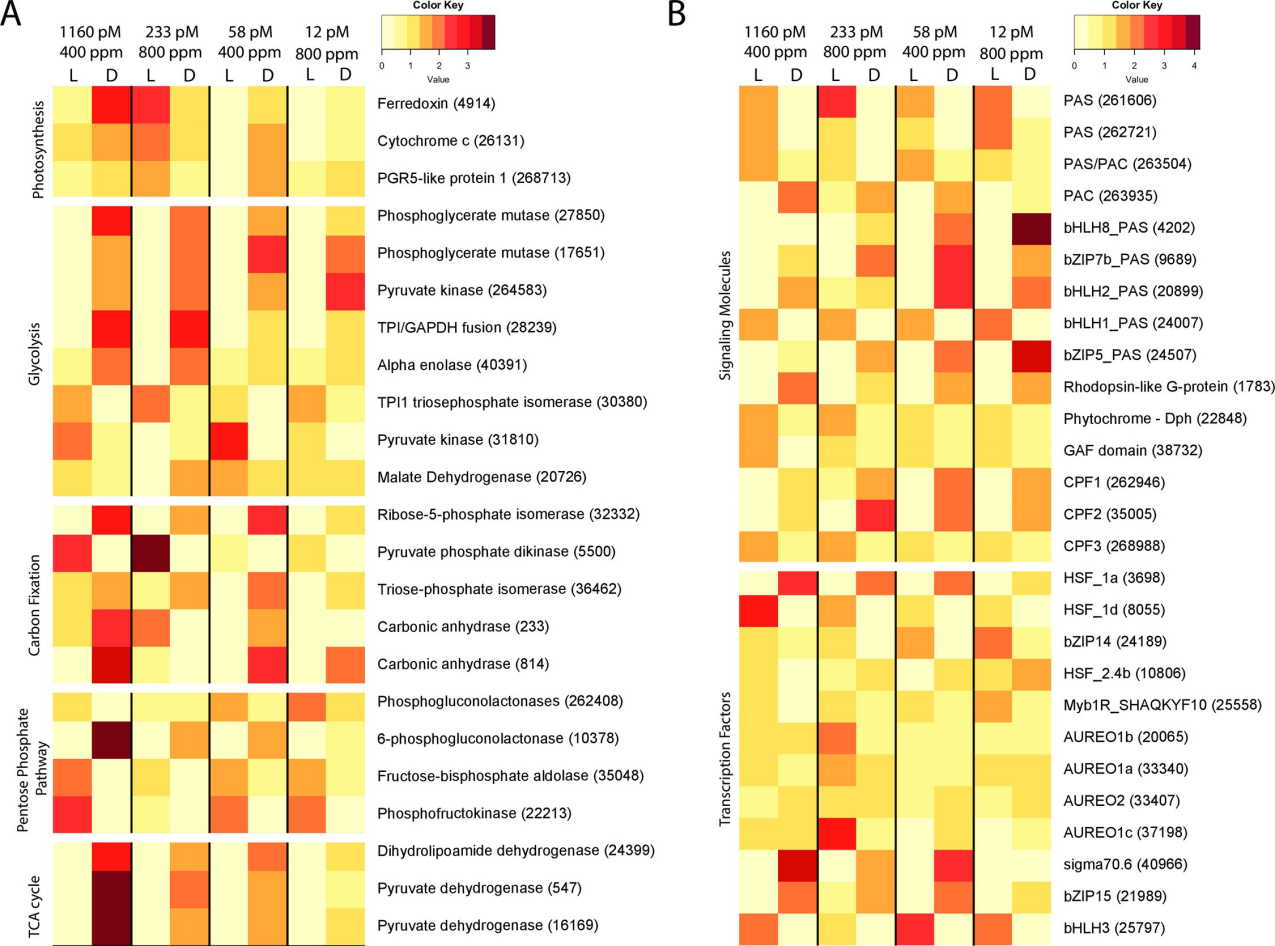
Heatmap of selected gene expression across all conditions. (A) Selected genes belonging to carbon related pathways. Each white line separates one pathway to the next. From top to bottom: Light-harvesting and Photosynthesis, Glycolysis, Carbon Fixation, Pentose Phosphate Pathway, and TCA cycle. (B) Selected genes belonging to the Signaling molecules (top) and the Transcription Factors (bottom).

Another interesting feature of this pathway was that in the light, average RPKM values increased at 233 pM, which coincided with the concomitant increase in CO_2_ concentration from 400 ppm to 800 ppm. In particular, nineteen genes that were day-expressed at 1160 pM, increased their expression during the day at 233 pM resulting in a higher log2FC (S4A Fig–indicated by a +, S6 and S7 Tables). Three genes (ferredoxin [Thaps3_4914], cytochrome c oxidase [Thaps3_26131], and a cyclic electron flow protein [Thaps3_268713]) also displayed night expression (or no diel regulation) at 1160 pM, 58 pM and 12 pM and day expression at 233 pM (Fig 4A).

A subset of genes within the oxidative phosphorylation pathway displayed a similar behavior. At 1160 pM, several genes that encode V-type ATPases were either expressed during the night or were not significantly expressed on a day: night cycle (expressed during both day and night). At 233 pM, these same genes were differentially expressed during the day (S4B Fig and S6 Table).

Transcriptional patterns of genes involved in the glycolysis pathway displayed less sensitivity to Fe’ than the other pathways, with about a third of the genes maintaining the same diel expression patterns at each Fe’. In diatoms, different components of the glycolysis pathway are localized in the cytoplasm, the plastid, and the mitochondrion [35]. Of the sixteen genes encoding mitochondrially-targeted enzymes (S4C Fig–indicated by a “M”), five (phosphoglycerate mutase 2 and 4 [Thaps3_27850 and Thaps3_17651], pyruvate kinase PK [Thaps3_264583], triose-phosphate isomerase [Thaps3_28239] and enolase-3 [Thaps3_40391]) were differentially transcribed at night (Fig 4A, S4C Fig and S6 Table). A malate dehydrogenase (Thaps3_20726), which is a component of the TCA cycle and also targeted to the mitochondrion, switched from non-significant diel expression at 1160 pM to differential transcription at night at 233 pM (Fig 4A). Of the fifteen genes encoding plastid-targeted proteins (S4C Fig–indicated by a “P”), only the genes encoding a pyruvate kinase and a triose phosphate isomerase maintained a constant transcription profile across all Fe’ (Fig 4A). No clear trend in timing of transcriptional patterns at each Fe’ were found for the thirty genes encoding glycolysis proteins retained in the cytoplasm (S4C Fig–indicated by a “C”). For example, those genes encoding cytoplasm-localized glycolytic enzymes shared between the pentose phosphate pathway and glycolysis were differentially transcribed during the day. Other genes were differentially transcribed during either the day (pyruvate phosphate dikinase, shared with the carbon fixation pathway) or the night (pyruvate kinases [Thaps3_40393 and Thaps3_22345], TPI [Thaps3_7229]).

The carbon fixation pathway maintained an overall constant diel expression at all Fe’ with ~50% of the genes expressed at night and ~10% expressed during the day (Fig 4A, S4D Fig). Despite this diel expression, transcript abundances decreased when Fe’ decreased overall from 1160 to 12 pM (S5 Fig), and only three genes within the pathway maintained consistent diel expression: a ribose phosphate isomerase (Thaps3_32332), a pyruvate dikinase (Thaps3_5500), and a triose phosphate isomerase (Thaps3_36462). The latter two genes are shared with the glycolytic pathway. The carbon fixation pathway appeared impacted by an increase in CO_2_. Two carbonic anhydrases (Thaps3_233, 814) displayed a change from night expression (or no diel expression) to a day expression only at 233 pM, where the CO_2_ was increased to 800 ppm. An additional subset of genes involved in carbon fixation were repressed at high CO_2_ levels regardless of the Fe' concentration: several carbonic anhydrases (Thaps3_31046, Thaps3_25840, Thaps3_34125, Thaps3_814), phosphoribulokinase (Thaps3_4376), fructose bisphosphate aldolase (Thaps3_428), ribose phosphate isomerase (Thaps3_32332) (S4D Fig, indicated by a *), while others increased at high CO_2_ (Thaps3_5500, Thaps3_22391, Thaps3_262006, Thaps3_262009) (S4D Fig, indicated by a **), indicating an effect of CO_2_ on gene-expression.

Maintenance of diel expression under the different Fe’ treatments was also apparent in the pentose phosphate pathway, which shares several genes with both glycolysis and carbon fixation pathways. Five genes maintained constant regulation across the different Fe’ concentrations and encode two phosphogluconolactonases (Thaps3_262408 during the day, Thaps3_10378 at night), a fructose-bisphosphate aldolase (Thaps3_35048), a phosphofructokinase (Thaps3_22213), and the aforementioned ribose-phosphate isomerase (Thaps3_32332) from the carbon fixation pathway. The phosphofructokinase and the fructose-bisphosphate aldolase were shared with glycolysis and differentially transcribed during the day (Fig 4A, S4E Fig).

Finally, glycolysis is connected to the tricarboxylic acid (TCA) cycle via the conversion of pyruvate (from glycolysis) to acetyl CoA (used in the TCA cycle). The three genes that maintained a night expression across all Fe’ in the TCA cycle were involved in this conversion, and thus overlapped with glycolysis: a dihydrolipoamide dehydrogenase (Thaps3_24399) and two pyruvate dehydrogenases (Thaps3_547, Thaps3_16169) (Fig 4A, S4F Fig).

Five genes from the nitrogen metabolism pathway maintained diel expression at night across all Fe’: a glutamine synthetase (Thaps3_26051), a carbamoyl-phosphate synthase (Thaps3_40323), two nitrate transporters (Thaps3_27414 and Thaps3_39592) and a nitrate reductase (Cyt b5: Thaps3_25299) (S4G Fig).

### Regulation of transcription: Sensing and signaling proteins and transcription factors

Differential transcription analysis was also conducted for the 250 transcription factors (TFs) identified by Rayko et al. [30] and the 150 signaling molecules, identified in part by Montsant et al. [31]. Transcripts were detected for genes encoding 225 out of 250 transcription factors, and 143 out of 150 signaling molecules (S4H–S4I Fig and S6 Table). Twelve genes were included in both pathways as they possessed both a DNA binding domain (bHLH or bZIP) and a signaling Per-Arnt-Sim (PAS) domain. The vast majority (75–80%) of genes encoding transcription factors and signaling molecules were differentially transcribed between day and night for at least one Fe’ level (Fig 3, S4H and S4I Fig and S6 Table). The proportion of these differentially transcribed genes decreased as Fe’ was reduced (Fig 3).

At the highest Fe’ level, a majority (~60%) of the genes encoding signaling molecules were differentially expressed at midday. This number decreased to 50% when Fe’ was reduced to 12 pM (Fig 4B; S4H Fig and S6 Table). Twenty-six of the genes encoding signaling molecules maintained differential transcription at all Fe’, with seventeen of these genes differentially expressed during the day. Ten of these day-expressed genes encoded the signaling domains PAS/PAC (PAC is a repeated PAS domain on the carboxyl side) (Thaps3_261606, Thaps3_262720, Thaps3_262721, Thaps3_263504, Thaps3_263935) or a PAS domain in conjunction with a DNA-binding (bHLH and bZIP) domain (Thaps3_4202, Thaps3_9689, Thaps3_20899, Thaps3_24007, Thaps3_24507). Additional genes that maintained differential day-night transcription included a gene encoding a rhodopsin-like G protein-coupled receptor (Thaps3_1783), a phytochrome (Dph; Thaps3_22848), a GAF domain (domain present in phytochromes cyclic GMP and others) (Thaps3_38732), commonly found in regulatory proteins, a cryptochrome/DNA-photolyase (CPF2; Thaps3_35005). Two other cryptochrome (CPF1; Thaps3_262946 and CPF3; Thaps3_268988) maintained diel expression for three Fe’ conditions. Together, these results reflect a maintenance of diel expression in light-signaling proteins (Fig 4B, S4H Fig).

In contrast to the signaling molecules, a majority (~55%) of diel regulated TFs were differentially transcribed at midnight at the two replete Fe’ levels, and this number increased at lower Fe’ concentrations, reaching ~70% at the two deplete Fe’ (Fig 4B, S4I Fig). Thirty TFs maintained differential transcription over the day/night cycle at all Fe’ and are likely essential for maintaining metabolic functions that require tight diel regulation (S4I Fig–indicated by a *). These included 10 heat shock factors (HSF), 5 bZIP TFs, and the 5 TFs with PAS domains (bHLH and bZIP families) (Fig 4B). Transcript abundance was significantly greater during the night for 17 and during the day for 12 genes (S4I Fig and S6 Table). Some of these genes were further modulated by Fe’. Among the genes differentially expressed during the night at all Fe’, HSF 1a (Thaps3_3698) transcript abundance decreased at lower Fe’ concentrations, while transcript abundance for the genes encoding bHLH8_PAS (Thaps3_4202) and bZIP5_PAS (Thaps3_24507) increased at lower Fe’ concentrations (Fig 4B). Among the genes differentially expressed during the day at all Fe’, HSF 1d (Thaps3_8055) abundance decreased at lower Fe’, while bZIP 14 (Thaps3_24189) increased at lower Fe’. One heat-shock factor (Thaps3_10806) was day-expressed at 1160 pM and night-expressed at all other Fe’. Another TF that maintained differential expression during the day was a second Myb1R protein that contains the SHAQKYF motif (Thaps3_25558) (Fig 4B), which has been associated with the circadian clock in plants [36]. Many of these TFs overlapped with TFs identified in Ashworth et al. [19] that maintained diel expression in cells in stationary phase. Expression of Aureochrome 1B and 1C was downregulated in the light at the two lower Fe’ concentrations (Fig 4B and S4I Fig). Aureochrome 1A maintained high level of expression during both day and night at the lowest Fe’ concentration, resulting in a loss of differential expression.

### Alternative splicing of transcripts: Example of selected isoforms

A subset of transcripts was detected that appeared to result from intron splicing that used both canonical and non-canonical splice sites. To explore the potential roles of transcripts of different lengths (isoforms) derived from the same gene, we used our dataset to develop new genes models. We then conducted differential expression analysis on the newly defined genes, selecting those genes with at least one isoform that significantly increased in abundance during the day and one isoform that significantly increased during the night. We focused on four genes that fulfilled these criteria and presented non-canonical splicing sites (S6 Fig). Only one of these genes encoded a protein of known function—triose phosphate isomerase 1 (TPI1), an enzyme shared by the glycolytic and the carbon fixation pathways.

Five isoforms were detected for the gene encoding TPI1 (sequences in S1 File). Three isoforms (isoforms 1, 2 and 4) encoded the known active site of the enzyme and closely resemble the gene model Thaps3_30380 (S6 Fig). Intron splicing for the other two isoforms (3 and 5) resulted in transcripts that did not encode the active site. The canonical splicing sites was gt/ag for those isoforms with the active site, while the non-canonical splicing sites for the isoforms without the active site was ac/cc. The non-canonical splice site resulted in the in-frame removal of an additional 120 nucleotides.

**Fig 5:**
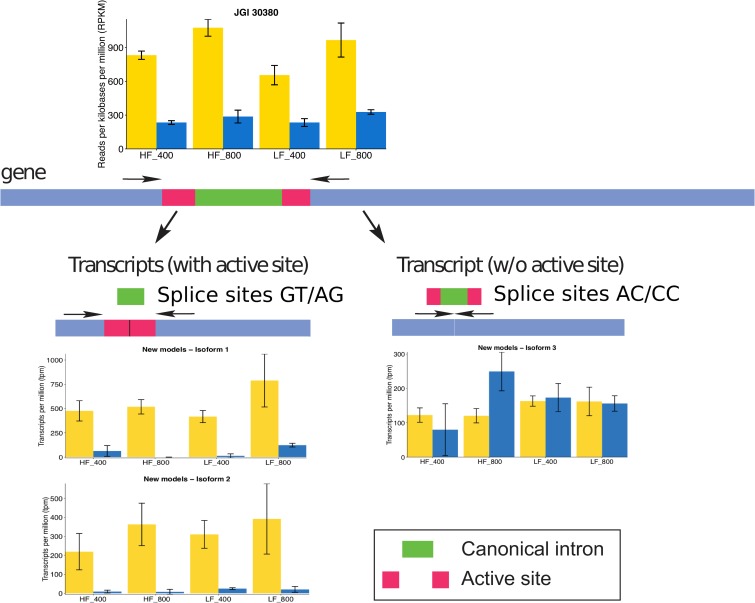
Schematic of intron splicing for gene JGI Thaps3_30380 and associated transcript abundance of the gene and its isoforms. The schematic illustrates the different splicing outcomes: splicing of the canonical intron leads to transcripts with the active site (Isoforms 1 and 2 both have the active site but differ in length by 71 amino acids); splicing of the non-canonical intron leads to an isoform without the active site. HF = high Fe addition, LF = low Fe addition. 400 and 800 are the CO_2_ levels (in ppm). The combination of Fe addition and CO_2_ levels result in four Fe’: 1160, 233, 58 and 12 pM. Bar colors indicate samples taken in the light (yellow) or in the dark (blue). Error bars represent standard deviation of the triplicates. Gene abundance is in counts per million or cpm (counts scaled by the number of fragments sequenced times one million) and isoform abundance is in transcript per million or tpm.

Isoforms 1 and 2, containing the active site, were differentially transcribed during the day at all Fe’ (Fig 5), consistent with our results based on the original JGI-produced gene model (S2 and S6 Tables). In contrast, transcript isoform 3 did not encode an active site and was not differentially transcribed between night and day at any Fe’ level (Fig 5). Few transcripts were detected for isoforms 4 and 5 under any condition (S7 Fig).

## Discussion

Our experimental design tested the interaction between iron availability and CO_2_ concentrations on the physiological and transcriptional responses of the model diatom *T*. *pseudonana* when grown on a light: dark cycle. Iron availability and CO_2_ concentrations are expected to co-vary in future ocean environments [10].

At a physiological level, Fe limitation was the main driver of the observed responses, although the overall changes were likely modulated by the light limiting conditions used in the experiments, and to some extent the increased CO_2_ concentration. For example, the low light levels (50 μE m^-2^ s^-1^) likely mitigated cell stress under the low Fe’ conditions resulting in relatively high Fv/Fm values, as seen in previous experiments [21]. Similar growth rates at 233 pM and 1160 pM indicated that the cells reached maximum growth rate at 233 pM, and that a negative impact was not observed by increasing Fe’ up to 1160 pM (Fig 1A). Higher cell yields were attained at lower Fe’ concentrations, reflecting a decrease of cell volume during the experiment (S1B and S1E Fig). The doubling of CO_2_ may have impacted cell physiology, as seen in the observed decrease of the normalized RFU/cell with increased CO_2_ (S1 Fig). This may reflect the balance between light harvesting and carbon fixation relative to photorespiration, and is in line with previous results in nitrate limited experiments [37].

At a transcriptional level, our experimental design illustrated the dominant influence of the light: dark cycle on transcriptional profiles, even under stressful environmental conditions (Fig 2). About 80% of all transcribed genes were differentially transcribed at either midday or midnight for at least one Fe’ condition. Sampling in the middle of the light and dark phases, allowed us to detect the influence of light on transcriptional patterns and identified strategies used by the cell in response to Fe limitation and, to a lesser extent, in response to CO_2_/pH change. Our design showed that Fe’ limitation induced a loss of diel regulation (Fig 3). Similar loss of diel expression was observed in a previous study under nitrogen limitation, an essential macronutrient [19].

At the two highest Fe’ treatments of 1160 pM and 233 pM, the cells displayed similar growth rates and yet different transcriptional profiles. In particular, the light profile at 233 pM was the most dissimilar to the other Fe’ treatments (Fig 2). This apparent discrepancy could be explained by the increased CO_2_ concomitant with the decreased Fe’. Indeed, the lower Fe availability for the photosynthetic machinery could be compensated by the decrease in energy expanded for carbon transport in the cell when CO_2_ is doubled. This would result in an overall similar growth rates for the two conditions, while displaying distinct transcriptional changes, as different genes and pathways would be mobilized. This is consistent with the transcriptional and physiological responses observed in our experiments. We do not expect this compensating effect between Fe’ and CO_2_ to be significant at lower Fe’ levels (58 and 12 pM). Indeed, at low Fe’, the rate limiting step of photosynthesis happens at the electron transport chain stage (the so-called “light reaction” of photosynthesis) rather than during carbon fixation (dark reaction), therefore precluding any positive impact of an increase of CO_2_ concentration.

More specifically, at a transcriptional level, when Fe’ decreased from 1160 (400 ppm CO_2_) to 233 pM (800 ppm), a subset of the photosynthesis and oxidative phosphorylation genes switched from differential expression at night to differential expression either during the day or similar expression levels during both the day and night (Fig 4A, S4 Fig and S6 Table). Some of these genes are involved in the electron transport chain (a plastoquinone and a ferredoxin for photosynthesis, five proton transport for oxidative phosphorylation), which is directly reliant on iron availability. Another subset of genes displayed the reverse behavior. A pyruvate kinase (Thaps3_31810), which catalyzes the last step of glycolysis, switched from differential transcription during the day at 1160 pM to differential transcription during the night at 233 pM, and a malate dehydrogenase (Thaps3_20726), which is a component of the TCA cycle, switched from non-significant diel expression at 1160 pM to differential transcription at night at 233 pM (Fig 4A, S4C Fig and S6 Table). These changes in transcriptional profiles between day and night may reflect a shift in photosynthetic energy allocation and a change in the carbon pools sizes from the low to high CO_2_ levels. Such shifts have been previously observed in diatoms and other phytoplankton with different growth rates [38] and are thought to be a key regulatory mechanism that allows the maintenance of relatively high growth rates for *T*. *pseudonana* at low light [39].

About half of the most differentially transcribed genes at the two deplete Fe' conditions were shared, denoting a consistent diel strategy at low Fe’. Limiting Fe’ also triggered the expression of genes involved in Fe uptake and assimilations. Two FTR1 iron permease genes, thought to facilitate transport of Fe across the cell membrane [40], are upregulated during both day and night at low Fe’ (S2 Table). They have previously been shown to be upregulated under Fe deplete conditions [41]. Interestingly, FTR1 requires a copper-requiring ferroxidase to function [41], and another gene upregulated at low Fe’ is a copper induced cell-surface protein. Other differentially transcribed genes encoded ABC transporters, heme peroxidases and cytochrome c-heme binding proteins. These genes may encode important tools for diatoms to compete with limiting Fe resources.

While Fe availability asserted the most notable effect on gene-expression levels, high CO_2_ levels affected the expression of genes involved in carbon fixation. Expression of several carbonic anhydrases (CAs) decreased at high CO_2_, regardless of Fe, while the expression of other CAs increased at high CO_2_ (Fig 4A, S4C Fig and S6 Table). The simultaneous change of CA activity could be due to the complex changes in the carbonate chemistry: the increase of CO_2_ results in a decrease of activity for some CAs (as confirmed in diatoms [42, 43], while the decrease in pH causes a decrease of carbonate ion which have been shown to co-limit Fe uptake in diatoms [11].

The total number of differentially expressed genes decreased with decreasing Fe’, which likely explains the KEGG pathway enrichment analysis that demonstrated the number of pathways significantly enriched in diel expressed genes increased with decreasing Fe’ (down to 58 pM). When ~60% of the genes are diely expressed at 1160 pM, comparatively few pathways can be considered significantly more enriched in diel expression. When overall diel expression decreases at low Fe’, the pathways that keep a majority of their genes diely expressed become more prominent. Additionally, at the lowest Fe’ level, all but two pathways enriched were also present at 58 pM, confirming the consistent diel strategy at low Fe’.

### Maintaining diel expression

Organisms gather information on their environment, such as nutrient status and light availability through light sensing and regulatory mechanisms. We found that over 70% of the genes involved in regulatory pathways displayed transcriptional patterns that were significantly different between the day and night at one or more Fe’, reiterating the importance of the diel cycle (Fig 4B, S4H and S4I Fig and S6 Table).

Diatoms possess multiple photoreceptors responsive to different wavelengths of light that belong to the cryptochrome (blue light), phytochrome (red/far-red light) and aureochrome (blue light) families (reviewed in Depauw et al. [44]). The blue light photoreceptor cryptochrome/photolyase1 (CPF1) constitutes an ancient type of photoreceptor, exerting DNA-repair activity as well as controlling the transcriptional regulation of nuclear encoded genes involved in light harvesting and pigment biosynthesis [45]. Phytochromes (Dph) enable the cell to sense and respond to red/far-red light [46], a wavelength range that does not penetrate deeply into the water column. Aureochromes contain a light-sensitive LOV domain and a bZIP DNA-binding domain. In the presence of blue light, these proteins can bind directly to DNA to act as a light-responsive transcription factor [47], thereby circumventing the use of conventional light signal transduction pathways.

The phytochrome and three of the four cryptochromes maintained day-night periodicity over three or more Fe’ conditions, indicating their importance for light signaling and possibly entrainment of the circadian clock (Fig 4B, S4H Fig and S6 Table). In contrast, the light-dependent transcription of Aureochrome 1B and 1C was downregulated in the light at the two lower Fe’ concentrations for both CO_2_ conditions (S4I Fig), and the light-dependent expression levels of these two photoreceptors are significantly correlated to Fe’ concentrations. The diel expression of Aureochrome 1A is also lost at lower Fe’ concentrations, in this case by maintaining high levels of expression during both night and day under Fe’ limitation. In the diatom *Phaeodactylum tricornutum*, Aueochrome 1A has been shown to control the light dependent onset of cell division [4]. Since both light and nutrients are essential for diatom cell cycle progression (Huysman et al. [48] and references therein), the loss of diel expression of these signaling molecule upon Fe’ limitation suggests that these light-sensitive proteins may play a part in aligning the cell cycle progression to the availability of important nutrients.

Aureochromes are not the only TFs that displayed diel rhythmicity. We found that thirty TFs maintained diel regulation across all Fe’ conditions, suggesting they regulate genes essential for basal metabolism. One category of TFs that maintained diel expression across all Fe’ conditions contained the Myb domain, which can be present as a single domain (Myb1R) or as two (Myb2R) or three (Myb3R) repeats. In plants, these TFs can regulate cellular morphogenesis and mediate signal transduction pathways [30]. We found that the gene encoding a Myb1R TF with a SHAQKYF motif was differentially transcribed during the day at all Fe’ (Fig 4B, S4I Fig and S6 Table). In *Arabidopsis*, a Myb1R protein with a SHAQKYF motif is a circadian clock-associated protein (CCA1) [36] that when bound to the light harvesting gene Lhcb1*3 promoter allows regulation by phytochrome. Maintenance of the same transcriptional profile across treatments suggests that this TF may similarly help maintain the circadian clock in *T*. *pseudonana* under stressful conditions. The *T*. *pseudonana* genome includes eight TFs that contain a PAS signal transducer motive in conjunction with a DNA-binding domain (either bHLH or bZIP) [30]. In *P*. *tricornutum*, homologues of the bHLH-PAS have been hypothesized to be an integral part of the diatom circadian clock, analogous to the bHLH-PAS proteins involved in the regulation of rhythmic processes in animals [49]. We found in *T*. *pseudonana*, that three out of four bHLH-PAS maintained day-night periodicity regardless of the Fe’ condition, displaying the strong diel transcriptional control of these genes, typical for clock-components. Moreover, two out of four bZIP-PAS also kept day-night periodicity over all Fe’ conditions, possibly adding novel candidates to the diatom internal circadian clock.

We compared our results with an earlier study in *T*. *pseudonana* by Ashworth et al. [19] that identified TFs with potential roles in regulating growth over the day: night cycle. In our study, we sampled at midday and midnight and in the Ashworth study, samples were taken at dusk and dawn, just before the onset of light or before the onset of dark. Neither study fully depicts the dynamics of transcription over the diel cycle, for which more frequent sampling is needed to accurately identify the timing of peak transcript abundance. This is exemplified by the transcription of the genes encoding HSF1a, sigma 70.6, bZIP15 and bHLH3, which here were each differentially upregulated at 6h into the dark phase across all Fe’ in our dataset. By contrast in Ashworth et al. [19], under nutrient replete conditions, peak transcript abundance for the HSF1a, sigma 70.6, and bZIP15 genes occurred 12h into the dark phase, whereas the bHLH3 peak occurred 12h into the light phase. Similarly, several TFs regulated at dawn or dusk in Ashworth et al. [19]–which the authors interpreted as an anticipation of the light or dark, respectively–reached peak transcript abundance at different times in our dataset. For example, two TFs with a PAS domain (bZIP7b_PAS and bHLH8_PAS) were transcribed at dawn in Ashworth *et al*, whereas they were transcribed at midnight in our experiments (Fig 4B, S4I Fig and S6 Table).

In the diatom *P*. *tricornutum*, the TF bZIP14 was identified as regulating the TCA cycle [50]. Its expression was also found to be diely regulated, as well as activated under nitrogen depletion, in three model diatoms: *T*. *pseudonana*, *P*. *tricornutum* as well as *Fragilariopsis cylindrus* [50]. Similarly, in our study, not only did bZIP14 maintain a strong diel regulation during the light period at all Fe’, transcript abundances during the day increased as Fe became more limiting (S4I Fig and S6 Table). Our results indicate a common theme of increased transcript abundance for bZIP14 under increasingly stressful conditions. It also suggests that diatoms may have retained or evolved similar strategies for regulating carbon pathways in nutrient limiting conditions.

### Maintaining diel expression: Temporal and spatial segregation

We commonly observed an asynchronous pattern of transcription across a given pathway relative to the day: night cycle. Glycolysis/gluconeogenesis displayed both temporal and spatial regulation. In this context, temporal is defined as an expression during either day or night, while spatial indicates the targeted organelle for a particular gene or pathway. This reversible pathway is used for production (glycolysis) or consumption (gluconeogenesis) of stored carbon and shares enzymes with other pathways such as carbon fixation, the pentose phosphate pathway and the TCA cycle. Moreover, many steps are reversible, with multiple isozymes [51], and different steps carried out in the cytosol, plastid, periplastid compartment as well as in the mitochondrion, a unique feature of diatoms [35]. In *T*. *pseudonana*, none of the organelles contain a full set of glycolytic enzymes: the cytosol contains all the enzymes from the preparatory or upper phase while the mitochondrion contains most enzymes of the lower or payoff phase. The plastid contains enzymes from both the upper and lower phase but lacks key enzymes that would allow either phase to run fully [35].

One of the glycolytic enzymes of interest is the cytosol-targeted TPI (Thaps3_30380). Based on the JGI-models, this gene was upregulated during the day at all Fe’. However, based on our splice-aware gene models, we detected several transcript isoforms for this gene: the isoforms that possessed the encoded active site accumulated during the day, whereas the isoform that lost the active site accumulated during both day and night (S7 Fig). The benefit of producing an isoform that would not result in an active enzyme is puzzling. Previous work in *P*. *tricornutum* suggested that alternative splicing was used to increase the diversity of transcripts to regulate gene expression in response to nutrient limitations [52]. The multiple isoforms may therefore be involved in the diel regulation of the cytosolic TPI under Fe stress.

In general, those glycolytic genes encoding mitochondria-targeted enzymes that maintained a strong periodicity at all Fe’ were preferentially upregulated at night (Fig 4A,S4C Fig and S6 Table). This suggests a coordinated use of this portion of the pathway with mitochondrial respiration and the TCA cycle, at a time when photosynthetic energy is not available. Indeed, the advantage of a payoff phase of glycolysis in the mitochondria is that NADH is produced in the organelle instead of requiring transport from the cytoplasm, an energy demanding process.

In the cytosol, most of the isozymes of the upper phase of glycolysis maintained differential transcription during the day at all Fe’ (Fig 4A, S4C Fig and S6 Table). Since the upper phase consumes energy in the form of ATP, running it during the day enables synchronization with photosynthetically produced energy. However, the transcription patterns of genes encoding pyruvate kinases (PK) stand out. Isozymes of this enzyme are found in the cytoplasm, mitochondria and plastid. Each of these genes maintained differential transcription at night at all Fe’ (with the notable exception of PK 31810 which switched from differential transcription during the day at 1160 pM to differential transcription during the night at 233 pM). The product of PK is pyruvate, which serves as an intermediate in the formation of acetyl-CoA for use in the TCA cycle in the mitochondria and fatty acid biosynthesis in the plastids. Synthesis of fatty acid is used for carbon storage, while the TCA cycle occurs at night to provide carbon intermediates for protein synthesis in the morning [38]. The midnight expression of all isozymes could therefore further reiterate coordination between the TCA cycle and fatty acid synthesis.

## Conclusion

Our study shows that, in concert with the physiological changes of reduced growth and downregulation of the photosynthetic machinery, the loss of day: night periodicity for a majority of genes and pathways is a major consequence of Fe limitation. Most of this loss was driven by a decrease of night expression. Overall, night profiles remained similar at all Fe’, whereas the day profiles showed a high variability, perhaps reflecting a more fine-tuned strategy of the cells during the time of photosynthesis. A large number of transcription factors and signaling molecules demonstrated strong resilience of their diel expression over Fe’, implying their important, yet largely unknown, role in maintaining cell basal metabolism. Our splice aware gene models uncovered the existence of previously uncharacterized non-canonical splice sites. In particular the example of triose phosphate isomerase, part of the glycolytic pathway, revealed that some of the transcript isoforms presented different diel expression patterns. These transcript isoforms may be part of a broader regulatory system used by the cells to maintain diel regulation of important pathways in conditions of environmental stress. Determining the extent to which transcript isoforms are used, and whether their usage varies among different environmental conditions, and among different diatoms, would help us understand how they contribute to the resilience of diatoms in fluctuating environments.

## Supporting information

S1 Fig Physiological characteristics throughout the experiment (exponential and stationary phase). Error bars represent standard deviation of triplicates. (A). Relative chlorophyll *a* (Relative Fluorescence Unit, RFU) over the course of the experiment. Arrows represent the time at midday and midnight when samples were taken for RNA. (B). Natural log of cell counts as determined by flow cytometry. (C). RFU normalized per cell over time (d). (D). Chlorophyll *a* (Chl*a*) normalized per cell (in μg/L) over time. NB: Chl*a* measurements were not taken for samples at 1160 pM. (E). Forward scatter (fsc) normalized to the beads fsc over time.
(PDF)

S2 Fig **Concentration (μmol/L) of nitrate (A), phosphate (B), and silicate (C) throughout the experiment (exponential and stationary)**. Errors bars represent standard deviation of triplicates.
(PDF)

S3 Fig Fv/Fm over time (exponential and stationary phase). Error bars represent standard deviation of triplicates. Shaded areas depict dark periods.
(PDF)

S4 Fig Heatmap of gene expression across all conditions within each KEGG-defined pathway. (A). Light-harvesting and Photosynthesis, (B). Oxidative phosphorylation, (C). Glycolysis, (D). Carbon fixation, (E). Pentose Phosphate Pathway, (F). TCA cycle, (G). N metabolism, (H). Signaling molecules (photoreceptors identified with an *), and (I). Transcription factors. An average RPKM value was calculated for each gene and each read count was normalized by this average (see S7 Table). Number next to gene names are JGI ID. Samples were either taken in the light at midday (L) or in the dark at midnight (D).
(PDF)

S5 Fig Normalized average of RPKM for each gene in each pathway. An average RPKM value was calculated for each gene in a pathway, and then each RPKM count was normalized by this average. For each Fe’ concentration, the average of all normalized expression value during the day (orange) and during the night (blue) was calculated. Error bars represent the 95% confidence interval of the normalized averages.
(PDF)

S6 Fig Identification of isoforms for selected genes. Screenshots from the IGV software for visualization of gene models and read coverage from our samples. The top blue model represents the JGI-produced model while the middle blue ones represent the splice-aware models derived from Stringtie. Bottom part is the read coverage from our samples. Pink and blue colors for the reads represent forward and reverse reads respectively. Grey curve above reads represent overall coverage of the gene. JGI 30380 corresponds to TPI1.
(PDF)

S7 Fig Expressions of the five isoforms associated with the jgi gene 30380 at the four Fe’ concentrations during the light phase (L, Yellow) or during the dark phase (D, Blue). Values of expression are in transcripts per million (tpm). Error bars represent standard deviation from the triplicates.
(PDF)

S1 File Nucleotide sequences of the five isoforms associated with the JGI gene 30380. (DOCX)

S1 Table Number of reads and base pairs (bp) before (raw) and after quality control (qc; which includes trimming) in each fastq file, and the experimental conditions associated. (XLSX)

S2 Table Table of the reads for each triplicate of each conditions. FL = 1160 pM, FH = 233 pM, L = 58 pM, and H = 12 pM. L and D indicates samples taken during the light (L) or during the dark (D).
(XLSX)

S3 Table Differential expression analysis of day vs night at each Fe’ concentration using edgeR. The columns are: jgi gene IDs, log of fold change (FC), log of counts per million (CPM), likelihood ratio (LR), p value, and false discovery rate (FDR). Only genes for which *p*<0.01 are represented.
(XLSX)

S4 Table DAVID (Database for Annotation, Visualization and Integrated Discovery) enrichment analysis of the genes consistently transcribed during the day or during the night at all Fe’ concentrations. The classification stringency was set on “high”. Highlighted in green are the pathways for which *p*<0.05 for enrichment.
(XLSX)

S5 Table Enrichment analysis for diel expression of KEGG pathways at different Fe’ concentrations. A Fisher exact test was performed for each of the 101 pathways, to determine the number of genes differentially expressed between the day and night time points. We then performed a Benjamini Hochburg procedure to remove false positives.
(XLSX)

S6 Table Differential expression analysis of day vs night at each Fe’ concentration using edgeR for genes of selected pathways. Summarized data are shown in Fig 2.
(XLSX)

S7 Table RPKM count of genes in metabolic pathways for each condition. An average RPKM value was calculated for each gene in a pathway and each read count was normalized by this average. For each Fe’ concentration, the average of all normalized expression value was calculated during the day and during the night. FL = 1160 pM, FH = 233 pM, L = 58 pM, and H = 12 pM. L and D indicates samples taken during the light (L) or during the dark (D).
(XLSX)
