# Supplementary figures and images for: Fe limitation decreases transcriptional regulation over the diel cycle in the model diatom Thalassiosira pseudonana
Source: PLoS One. 2019 Sep 11;14(9):e0222325. doi: 10.1371/journal.pone.0222325 (PMC6738920; doi:10.1371/journal.pone.0222325)

**A**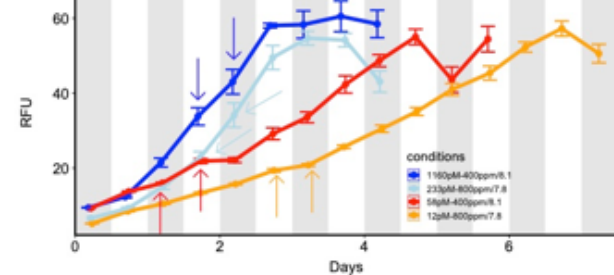**B**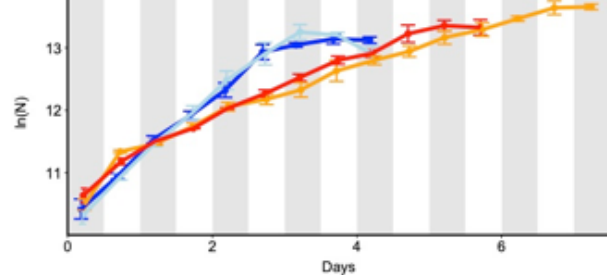**C**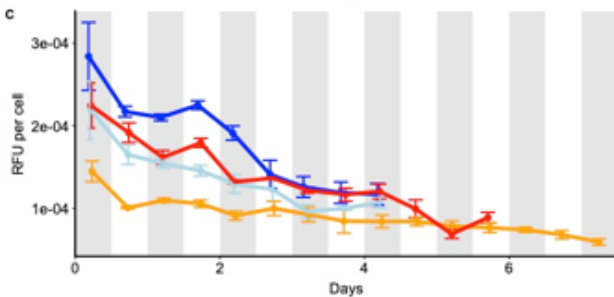**D**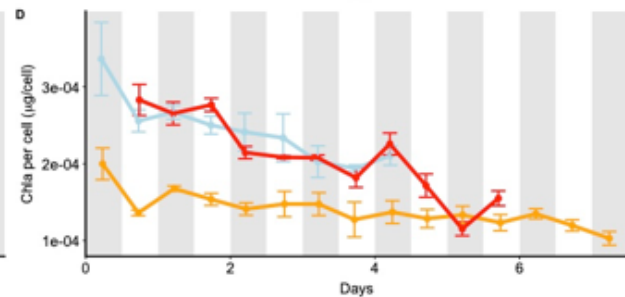**E**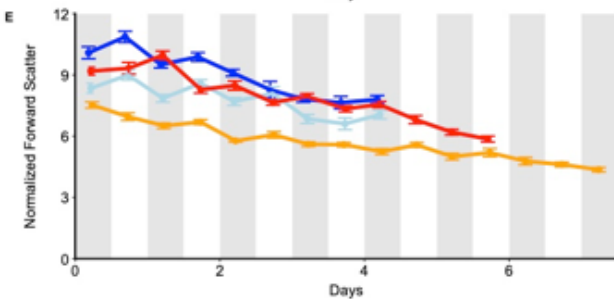

Supplement: S1 Fig — Error bars represent standard deviation of triplicates. (A). Relative chlorophyll a (Relative Fluorescence Unit, RFU) over the course of the experiment. Arrows represent the time at midday and midnight when samples were taken for RNA. (B). Natural log of cell counts as determined by flow cytometry. (C). RFU normalized per cell over time (d). (D). Chlorophyll a (Chla) normalized per cell (in μg/L) over time. NB: Chla measurements were not taken for samples at 1160 pM. (E). Forward scatter (fsc) normalized to the beads fsc over time. (PDF) [file pone.0222325.s001.pdf]

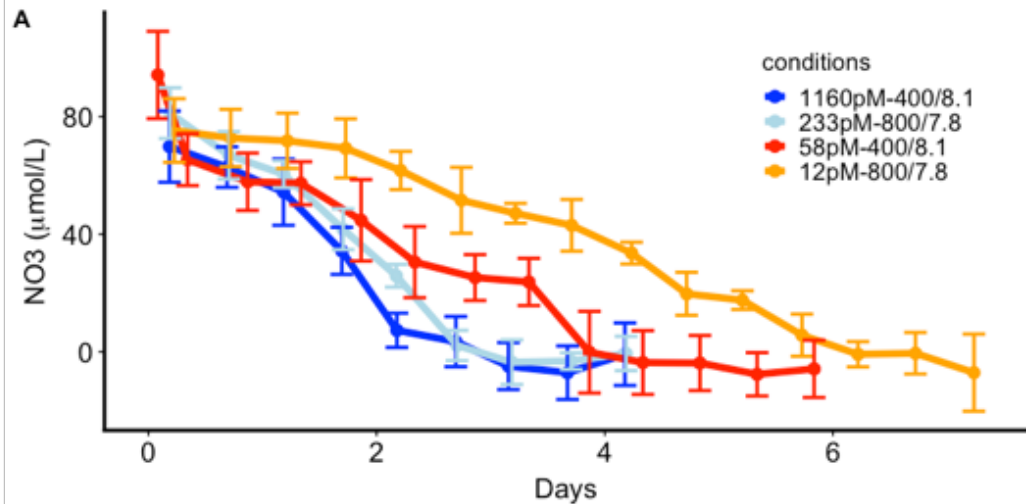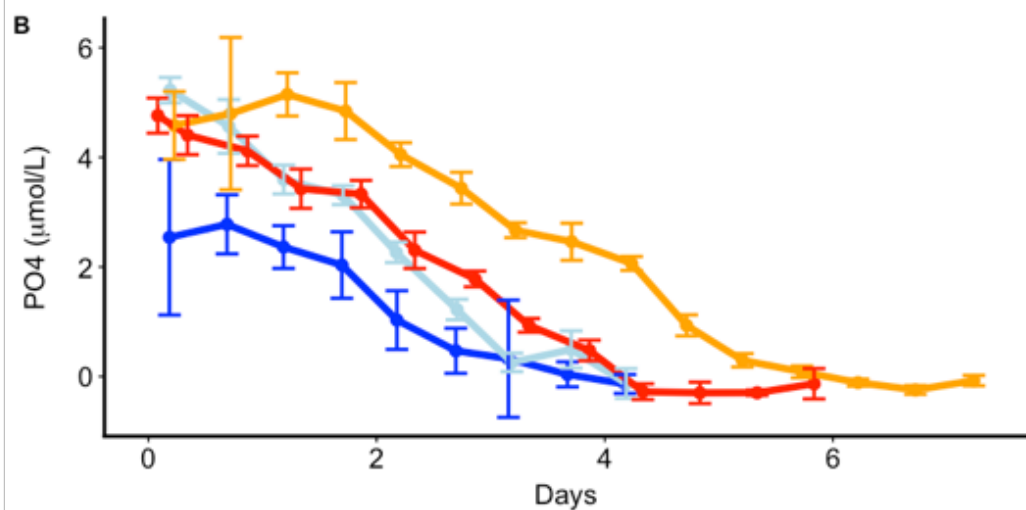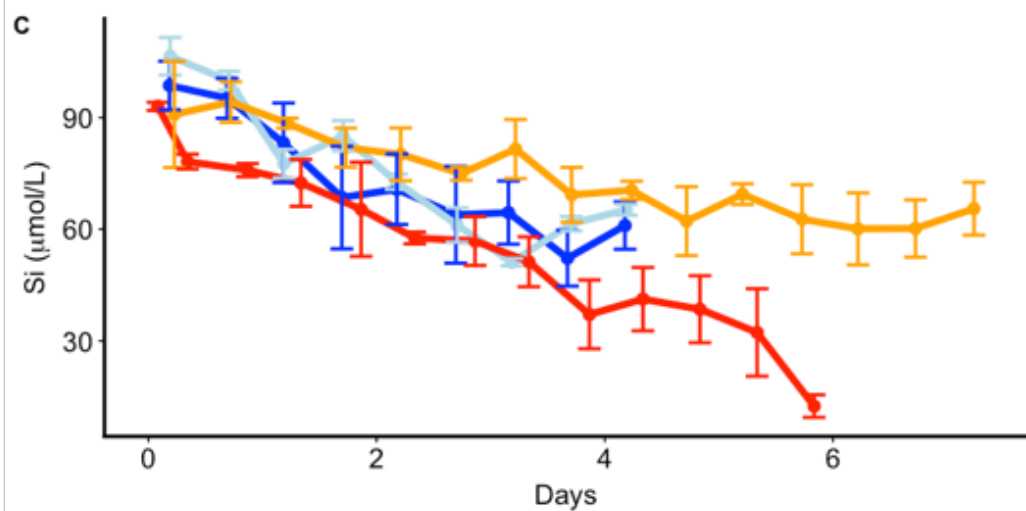

Supplement: S2 Fig — Concentration (μmol/L) of nitrate (A), phosphate (B), and silicate (C) throughout the experiment (exponential and stationary). Errors bars represent standard deviation of triplicates. (PDF) [file pone.0222325.s002.pdf]

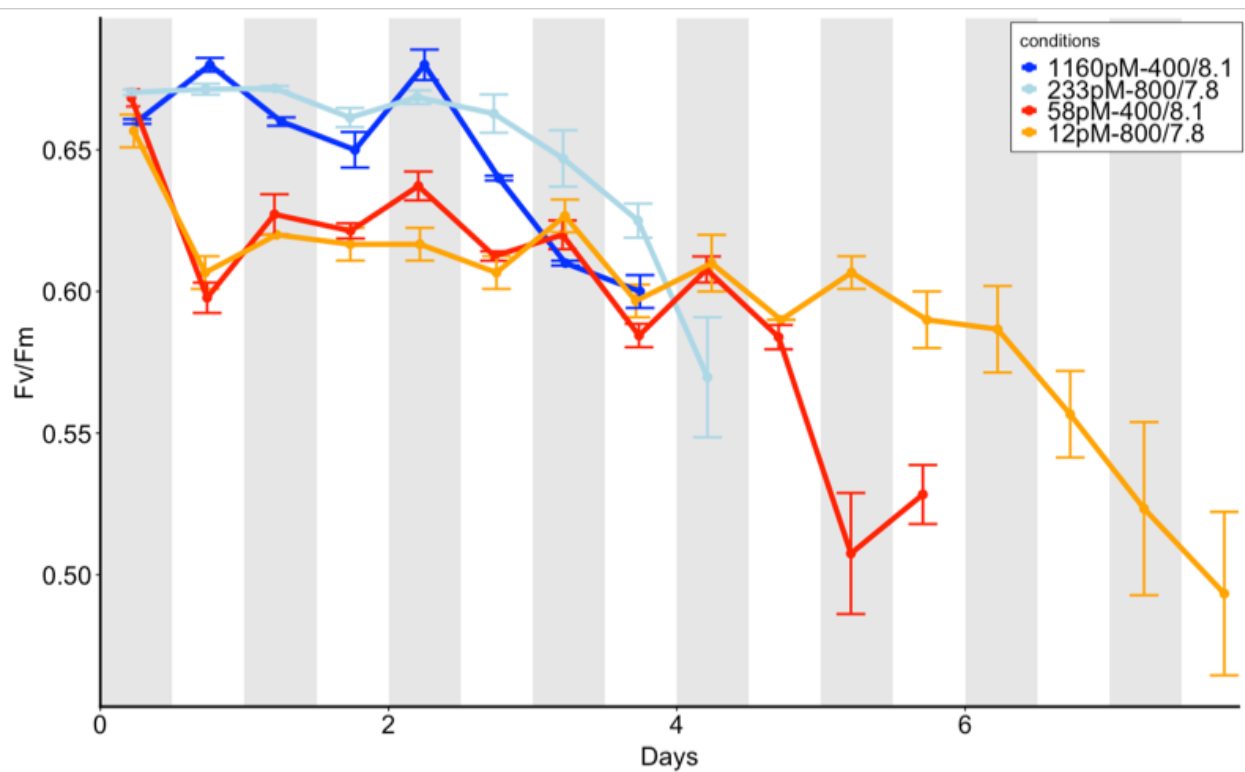

Supplement: S3 Fig — Error bars represent standard deviation of triplicates. Shaded areas depict dark periods. (PDF) [file pone.0222325.s003.pdf]

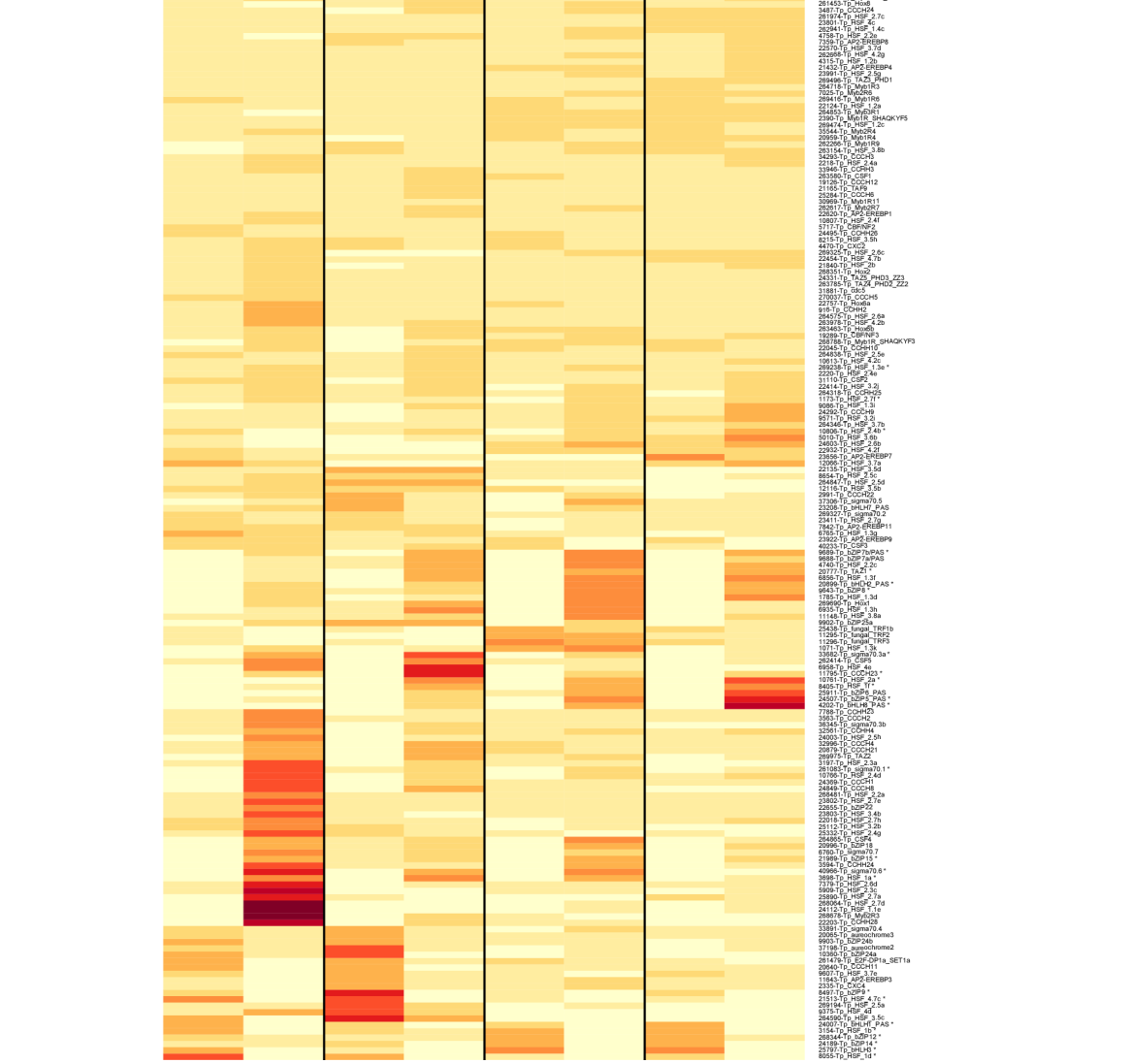

Supplement: S4 Fig — (A). Light-harvesting and Photosynthesis, (B). Oxidative phosphorylation, (C). Glycolysis, (D). Carbon fixation, (E). Pentose Phosphate Pathway, (F). TCA cycle, (G). N metabolism, (H). Signaling molecules (photoreceptors identified with an *), and (I). Transcription factors. An average RPKM value was calculated for each gene and each read count was normalized by this average (see S7 Table). Number next to gene names are JGI ID. Samples were either taken in the light at midday (L) or in the dark at midnight (D). (PDF) [file pone.0222325.s004.pdf]

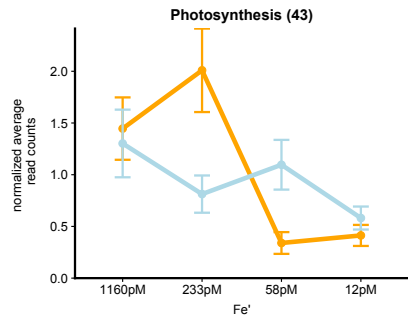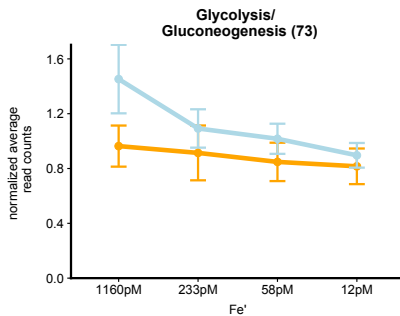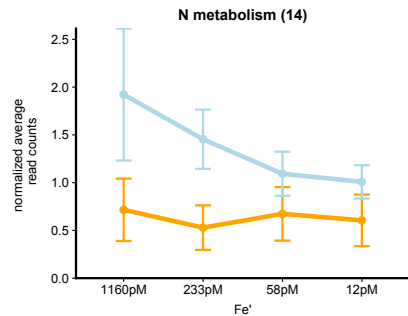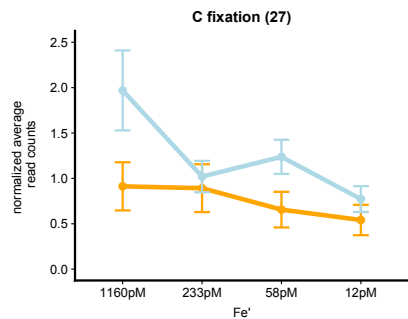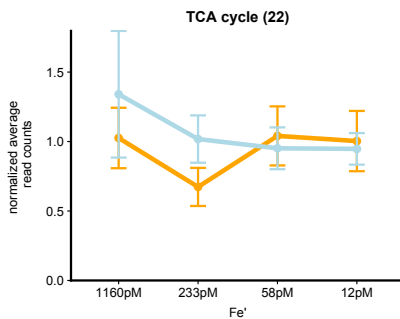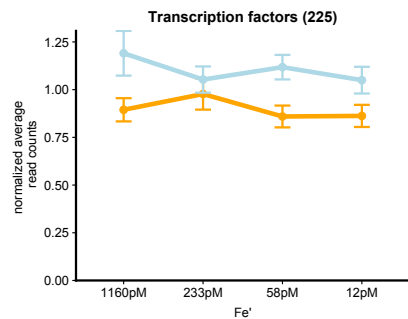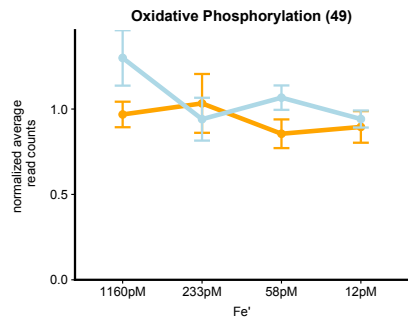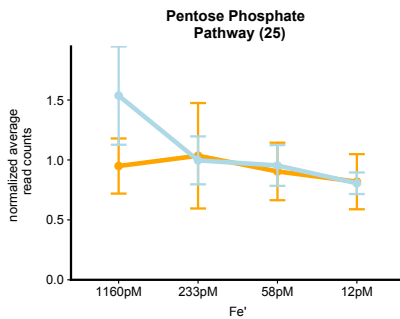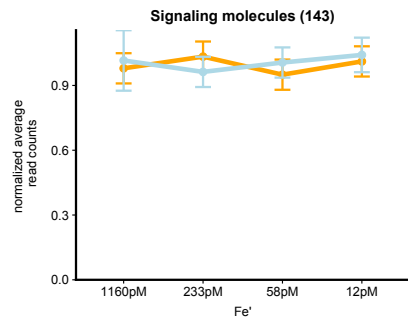

Supplement: S5 Fig — An average RPKM value was calculated for each gene in a pathway, and then each RPKM count was normalized by this average. For each Fe’ concentration, the average of all normalized expression value during the day (orange) and during the night (blue) was calculated. Error bars represent the 95% confidence interval of the normalized averages. (PDF) [file pone.0222325.s005.pdf]

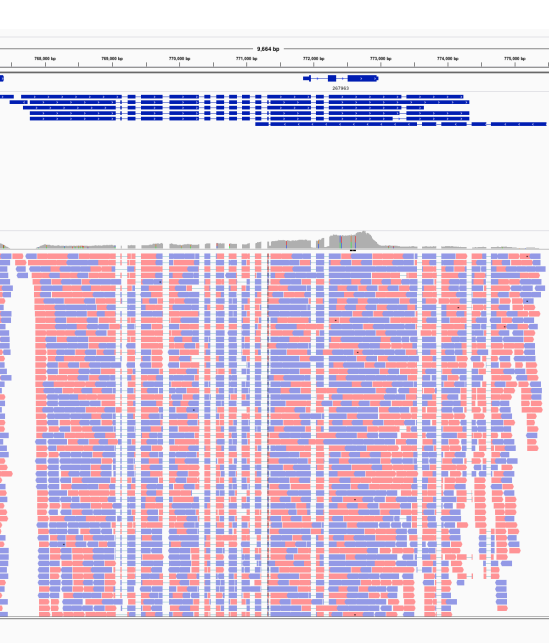

Jgi 267963

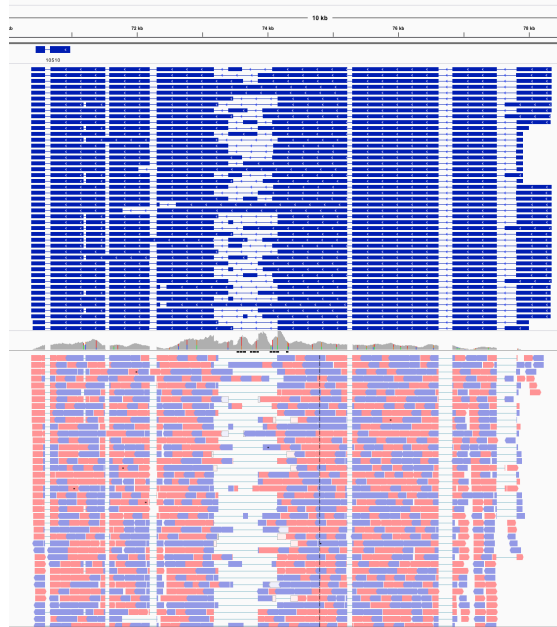

Jgi 10510

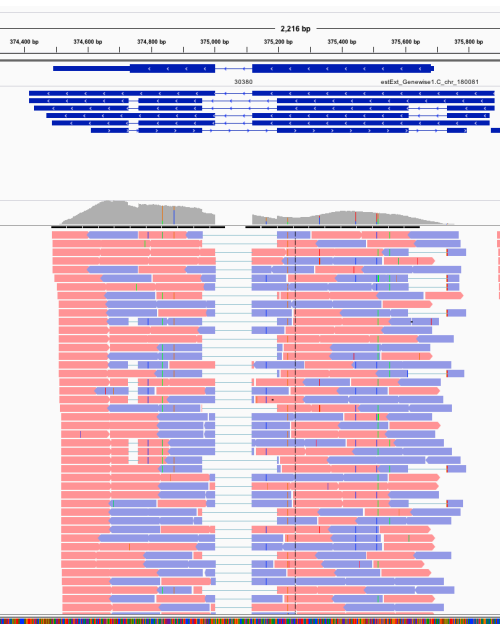

Jgi 30380

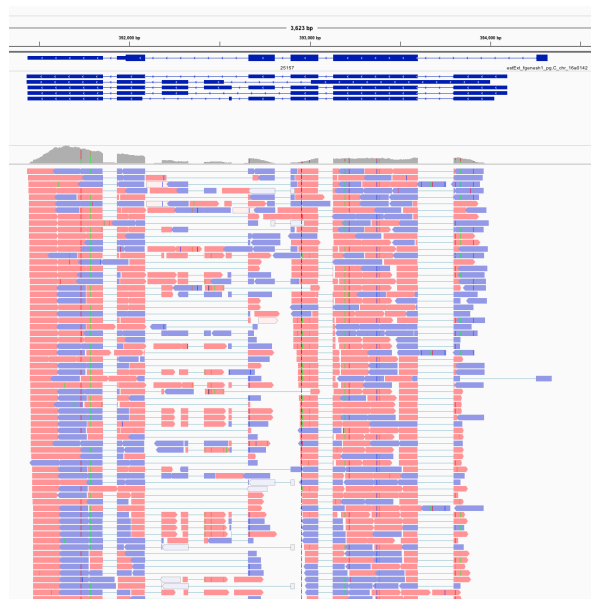

Jgi 25157

Supplement: S6 Fig — Screenshots from the IGV software for visualization of gene models and read coverage from our samples. The top blue model represents the JGI-produced model while the middle blue ones represent the splice-aware models derived from Stringtie. Bottom part is the read coverage from our samples. Pink and blue colors for the reads represent forward and reverse reads respectively. Grey curve above reads represent overall coverage of the gene. JGI 30380 corresponds to TPI1. (PDF) [file pone.0222325.s006.pdf]

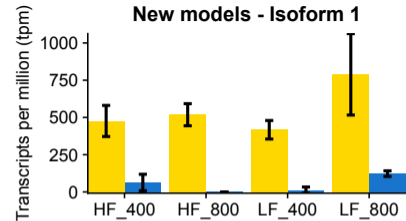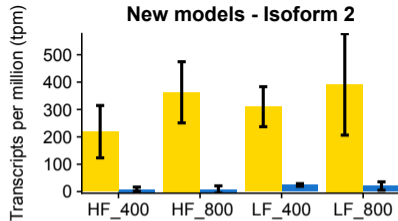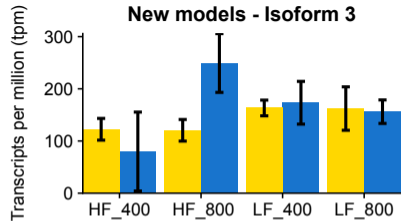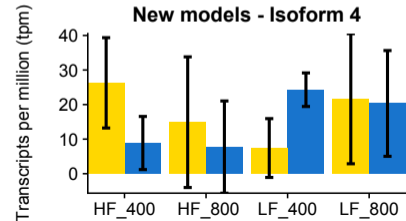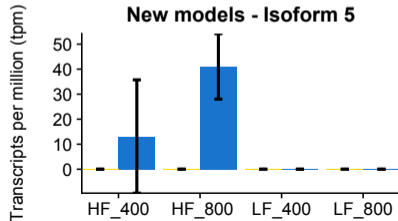

Supplement: S7 Fig — Values of expression are in transcripts per million (tpm). Error bars represent standard deviation from the triplicates. (PDF) [file pone.0222325.s007.pdf]
