# Supplementary material for: Fe limitation decreases transcriptional regulation over the diel cycle in the model diatom Thalassiosira pseudonana
Source: PLoS One. 2019 Sep 11;14(9):e0222325. doi: 10.1371/journal.pone.0222325 (PMC6738920; doi:10.1371/journal.pone.0222325)
Supplement: S1 File — (DOCX) [file pone.0222325.s008.docx]

>New models - Isoform 1

TGTGAAGACGCTTGTCGGTGGTTGTGGCGCGCCCGTCTGGATACGCGACGTCTTGGGCAGCTGGCGGTGTGCGTGAGTTCGTGTTGCTGCACCTTCAGCTTCCTCCGCCTCGCCTCGTCACTCGTCACAGCAACACAAAAGCCTTCTGCGCTTGCCCATAATTCAGCCAGCCAGCTCTTGATATCAATGGGAAATACCACCATGTCCTTCTCTTCTGCCTGCAAACGCCCCTCTGGCTCCTCCAAGGGCACTCCCGCCCCCGAAGGCGAACGCAAGTACCTCATCGGCGGCAACTGGAAGTGCAACGGAACCATCGCCTCCCTTGAAAAGATCATCTCTGAATTCAACGCTGCCGGCCCCATCCCCGCCAACGCCGAGGTGGTCATCGGATGCCCTGCCATGCACATTCCCATCGCCCTCATGTCCCTCCGTGACGATATCGAAATTGCCGCTCAGAACTGCAGCCTTACCGGATGCGGAGCTTACACTGGCGAAATTGCCGCTTCTCAACTCAAAGATATGGGTGTGACGTGGGTGATTCTTGGACATTCGGAGCGTCGTGAGGGATTTGGAATGGCTGGCGAGGATAGCGAGTTGGTGGCCAAGAAGACGAAGAAGGCCATTGAGGAGGGGTTGAAGGTCATGTTCTGTATCGGCGAGAAGAAGGAGGAGAGGGAGGCGGGTACTACCATGGATGTTTGTGCCAGTCAGTTGAAGCCTGCTGCTGATATTCTTAGCAAGGAGGATTGGGCCAACGTTTCCATTGCCTACGAACCAGTATGGGCAATCGGAACCGGCCTCACCGCCACCCCCGAGATGGCACAAGAAACGCACGCCAACATCCGTGCTTGGGTGGCCGAGAACGTTGGACAGGATGTTGCCGACGCTGTTCGTATCCAATACGGTGGAAGTATGAAGGGAGCAAATGCCGCTGATCTTTTGGCCCAGGCTGATATCGATGGAGGTCTTATCGGAGGTGCTTCCTTGACTATGGACTTCTTTAACTGTGTCAACGGTGTTCCCTCTCCTTAAGTTGTGTGCTAGAAGTGAACAGTTGGTGTAAGGAGTGAAGATCTGAATTCGACGAAACAACAGCCTTTCTTCACAGCAGTATAGTTTGGTTGCCACAAGAGTACGATGCAATAATTTGCACCCCCTTTCGAAGGAATGATGTCGCTGGCAGTGTCTGAGATGGGAAACGCAAGACGAGCGGCAGGTTTCTTCTGTAGAGAGCAAGTAGAGAGTAGCTTTGAACTAAATGAAAGTTATATTCAATGGATTGTGAATGCCTACCATCATTCAACTCGTGCTAGTCTGCCGTGATGATGATCGGCATCGTACAAGCAATA

>New models – Isoform 2

CGGTGGTTGTGGCGCGCCCGTCTGGATACGCGACGTCTTGGGCAGCTGGCGGTGTGCGTGAGTTCGTGTTGCTGCACCTTCAGCTTCCTCCGCCTCGCCTCGTCACTCGTCACAGCAACACAAAAGCCTTCTGCGGCAAGTACCTCATCGGCGGCAACTGGAAGTGCAACGGAACCATCGCCTCCCTTGAAAAGATCATCTCTGAATTCAACGCTGCCGGCCCCATCCCCGCCAACGCCGAGGTGGTCATCGGATGCCCTGCCATGCACATTCCCATCGCCCTCATGTCCCTCCGTGACGATATCGAAATTGCCGCTCAGAACTGCAGCCTTACCGGATGCGGAGCTTACACTGGCGAAATTGCCGCTTCTCAACTCAAAGATATGGGTGTGACGTGGGTGATTCTTGGACATTCGGAGCGTCGTGAGGGATTTGGAATGGCTGGCGAGGATAGCGAGTTGGTGGCCAAGAAGACGAAGAAGGCCATTGAGGAGGGGTTGAAGGTCATGTTCTGTATCGGCGAGAAGAAGGAGGAGAGGGAGGCGGGTACTACCATGGATGTTTGTGCCAGTCAGTTGAAGCCTGCTGCTGATATTCTTAGCAAGGAGGATTGGGCCAACGTTTCCATTGCCTACGAACCAGTATGGGCAATCGGAACCGGCCTCACCGCCACCCCCGAGATGGCACAAGAAACGCACGCCAACATCCGTGCTTGGGTGGCCGAGAACGTTGGACAGGATGTTGCCGACGCTGTTCGTATCCAATACGGTGGAAGTATGAAGGGAGCAAATGCCGCTGATCTTTTGGCCCAGGCTGATATCGATGGAGGTCTTATCGGAGGTGCTTCCTTGACTATGGACTTCTTTAACTGTGTCAACGGTGTTCCCTCTCCTTAAGTTGTGTGCTAGAAGTGAACAGTTGGTGTAAGGAGTGAAGATCTGAATTCGACGAAACAACAGCCTTTCTTCACAGCAGTATAGTTTGGTTGCCACAAGAGTACGATGCAATAATTTGCACCCCCTTTCGAAGGAATGATGTCGCTGGCAGTGTCTGAGATGGGAAACGCAAGACGAGCGGCAGGTTTCTTCTGTAGAGAGCAAGTAGAGAGTAGCTTTGAACTAAATGAAAGTTATATTCAATGGATTGTGAATGCCTACCA

>New models – Isoform 3

TGTGAAGACGCTTGTCGGTGGTTGTGGCGCGCCCGTCTGGATACGCGACGTCTTGGGCAGCTGGCGGTGTGCGTGAGTTCGTGTTGCTGCACCTTCAGCTTCCTCCGCCTCGCCTCGTCACTCGTCACAGCAACACAAAAGCCTTCTGCGCTTGCCCATAATTCAGCCAGCCAGCTCTTGATATCAATGGGAAATACCACCATGTCCTTCTCTTCTGCCTGCAAACGCCCCTCTGGCTCCTCCAAGGGCACTCCCGCCCCCGAAGGCGAACGCAAGTACCTCATCGGCGGCAACTGGAAGTGCAACGGAACCATCGCCTCCCTTGAAAAGATCATCTCTGAATTCAACGCTGCCGGCCCCATCCCCGCCAACGCCGAGGTGGTCATCGGATGCCCTGCCATGCACATTCCCATCGCCCTCATGTCCCTCCGTGACGATATCGAAATTGCCGCTCAGAACTGCAGCCTTACCGGATGCGGAGCTTACACTGGCGAAATTGCCGCTTCTCAACTCAAAGATATGGGTGTGACGTGGGTGATTCTTGGACATTCGGAGCGTCGTGAGGGATTTGGAATGGCTGGCGAGGATAGCGAGTTGGTGGCCAAGAAGACGAAGAAGGCCATTGAGGAGGGGTTGAAGGTCATGTTCTGTATCGGCGAGAAGAAGGAGGAGAGGGAGGCGGGTGCCACCCCCGAGATGGCACAAGAAACGCACGCCAACATCCGTGCTTGGGTGGCCGAGAACGTTGGACAGGATGTTGCCGACGCTGTTCGTATCCAATACGGTGGAAGTATGAAGGGAGCAAATGCCGCTGATCTTTTGGCCCAGGCTGATATCGATGGAGGTCTTATCGGAGGTGCTTCCTTGACTATGGACTTCTTTAACGTGCTAGAAGTGAACAGTTGGTGTA

AGGAGTGAAGATCTGAATTCGACGAAACAACAGCCTTTCTTCACAGCAGTATAGTTTGGTTGCCACAAGAGTACGATGCAATAATTTGCACCCCCTTTCGAAGGAATGATGTCGCTGGCAGTGTCTGAGATGGGAAACGCAAGACGAGCGGCAGGTTTCTTCTGTAGAGAGCAAGTAGAGAGTAGCTTTGAACTAAATGAAAGTTATATTCAATGGATTGTGAATGCCTACCATCATTCAACTCGTGCTAGTCTGCCGTGATGATGATCGGCATCGTACAAGCAATA

> New models – Isoform 4

CGGTGGTTGTGGCGCGCCCGTCTGGATACGCGACGTCTTGGGCAGCTGGCGGTGTGCGTGAGTTCGTGTTGCTGCACCTTCAGCTTCCTCCGCCTCGCCTCGTCACTCGTCACAGCAACACAAAAGCCTTCTGCGCTTGCCCATAATTCAGCCAGCCAGCTCTTGATATCAATGGGAAATACCACCATGTCCTTCTCTTCTGCCTGCAAACGCCCCTCTGGCTCCTCCAAGGGCACTCCCGCCCCCGAAGGCGAACGCAAGTACCTCATCGGCGGCAACTGGAAGTGCAACGGAACCATCGCCTCCCTTGAAAAGATCATCTCTGAATTCAACGCTGCCGGCCCCATCCCCGCCAACGCCGAGGTGGTCATCGGATGCCCTGCCATGCACATTCCCATCGCCCTCATGTCCCTCCGTGACGATATCGAAATTGCCGCTCAGAACTGCAGCCTTACCGGATGCGGAGCTTACACTGGCGAAATTGCCGCTTCTCAACTCAAAGATATGGGTGTGACGTGGGTGATTCTTGGACATTCGGAGCGTCGTGAGGGATTTGGAATGGCTGGCGAGGATAGCGAGTTGGTGGCCAAGAAGACGAAGAAGGCCATTGAGGAGGGGTTGAAGGTCATGTTCTGTATCGGCGAGAAGAAGGAGGAGAGGGAGGCGGGTACTACCATGGATGTTTGTGCCAGTCAGTTGAAGCCTGCTGCTGATATTCTTAGCAAGGAGGATTGGGCCAACGTTTCCATTGCCTACGAACCAGTATGGGCAATCGGAACCGGCCTCACCGCCACCCCCGAGATGGCACAAGAAACGCACGCCAACATCCGTGCTTGGGTGGCCGAGAACGTTGGACAGGATGTTGCCGACGCTGTTCGTATCCAATACGGTGGAAGTATGAAGGGAGCAA

ATGCCGCTGATCTTTTGGCCCAGGCTGATATCGATGGAGGTCTTATCGGAGGTGCTTCCTTGACTATGGACTTCTTTAACGTGCTAGAAGTGAACAGTTGGTGTAAGGAGTGAAGATCTGAATTCGACGAAACAACAGCCTTTCTTCACAGCAGTATAGTTTGGTTGCCACAAGAGTACGATGCAATAATTTGCACCCCCTTTCGAAGGAATGATGTCGCTGGCAGTGTCTGAGATGGGAAACGCAAGACGAGCGGCAGGTTTCTTCTGTAGAGAGCAAGTAGAGAGTAGCTTTGAACTAAATGAAAGTTATATTCAATGG

>New models – Isoform 5

TGTGAAGACGCTTGTCGGTGGTTGTGGCGCGCCCGTCTGGATACGCGACGTCTTGGGCAGCTGGCGGTGTGCGTGAGTTCGTGTTGCTGCACCTTCAGCTTCCTCCGCCTCGCCTCGTCACTCGTCACAGCAACACAAAAGCCTTCTGCGGCAAGTACCTCATCGGCGGCAACTGGAAGTGCAACGGAACCATCGCCTCCCTTGAAAAGATCATCTCTGAATTCAACGCTGCCGGCCCCATCCCCGCCAACGCCGAGGTGGTCATCGGATGCCCTGCCATGCACATTCCCATCGCCCTCATGTCCCTCCGTGACGATATCGAAATTGCCGCTCAGAACTGCAGCCTTACCGGATGCGGAGCTTACACTGGCGAAATTGCCGCTTCTCAACTCAAAGATATGGGTGTGACGTGGGTGATTCTTGGACATTCGGAGCGTCGTGAGGGATTTGGAATGGCTGGCGAGGATAGCGAGTTGGTGGCCAAGAAGACGAAGAAGGCCATTGAGGAGGGGTTGAAGGTCATGTTCTGTATCGGCGAGAAGAAGGAGGAGAGGGAGGCGGGTGCCACCCCCGAGATGGCACAAGAAACGCACGCCAACATCCGTGCTTGGGTGGCCGAGAACGTTGGACAGGATGTTGCCGACGCTGTTCGTATCCAATACGGTGGAAGTATGAAGGGAGCAAATGCCGCTGATCTTTTGGCCCAGGCTGATATCGATGGAGGTCTTATCGGAGGTGCTTCCTTGACTATGGACTTCTTTAACGTGCTAGAAGTGAACAGTTGGTGTAAGGAGTGAAGATCTGAATTCGACGAAACAACAGCCTTTCTTCACAGCAGTATAGTTTGGTTGCCACAAGAGTACGATGCAATAATTTGCACCCCCTTTCGAAGGAATGATGTCGCTGGCAGTGTCTGAGATGGGAAACGCAAGACGAGCGGCAGGTTTCTTCTGTAGAGAGCAAGTAGAGAGTAGCTTTGAACTAAATGAAAGTTATATTCAATGGATTGTGAATGCCTACCATCATTCAACTCGTGCTAGTCTGCCGTGATGATGATCGGC
